# Physicochemical tools for studying virus interactions with targeted cell membranes in a molecular and spatiotemporally resolved context

**DOI:** 10.1007/s00216-021-03510-5

**Published:** 2021-09-07

**Authors:** Marta Bally, Stephan Block, Fredrik Höök, Göran Larson, Nagma Parveen, Gustaf E. Rydell

**Affiliations:** 1 Department of Clinical Microbiology & Wallenberg Centre for Molecular Medicine, Umeå University, 901 85 Umeå, Sweden; 2 Department of Chemistry and Biochemistry, Freie Universität Berlin, 14195 Berlin, Germany; 3 Department of Physics, Chalmers University of Technology, 412 96 Gothenburg, Sweden; 4 Department of Laboratory Medicine, Sahlgrenska Academy at the University of Gothenburg, Sahlgrenska University Hospital, Bruna Stråket 16, 413 45 Gothenburg, Sweden; 5 Department of Chemistry, Indian Institute of Technology Kanpur, Kanpur, 208016 India; 6 Department of Infectious Diseases, Sahlgrenska Academy at the University of Gothenburg, 413 46 Gothenburg, Sweden

**Keywords:** Virus protein to host membrane interactions, Quartz crystal microbalance with dissipation, Dynamic force spectroscopy, Total internal reflection fluorescence microscopy, Equilibrium fluctuation analysis, Single particle tracking

## Abstract

The objective of this critical review is to provide an overview of how emerging bioanalytical techniques are expanding our understanding of the complex physicochemical nature of virus interactions with host cell surfaces. Herein, selected model viruses representing both non-enveloped (simian virus 40 and human norovirus) and enveloped (influenza A virus, human herpes simplex virus, and human immunodeficiency virus type 1) viruses are highlighted. The technologies covered utilize a wide range of cell membrane mimics, from supported lipid bilayers (SLBs) containing a single purified host membrane component to SLBs derived from the plasma membrane of a target cell, which can be compared with live-cell experiments to better understand the role of individual interaction pairs in virus attachment and entry. These platforms are used to quantify binding strengths, residence times, diffusion characteristics, and binding kinetics down to the single virus particle and single receptor, and even to provide assessments of multivalent interactions. The technologies covered herein are surface plasmon resonance (SPR), quartz crystal microbalance with dissipation (QCM-D), dynamic force spectroscopy (DFS), total internal reflection fluorescence (TIRF) microscopy combined with equilibrium fluctuation analysis (EFA) and single particle tracking (SPT), and finally confocal microscopy using multi-labeling techniques to visualize entry of individual virus particles in live cells. Considering the growing scientific and societal needs for untangling, and interfering with, the complex mechanisms of virus binding and entry, we hope that this review will stimulate the community to implement these emerging tools and strategies in conjunction with more traditional methods. The gained knowledge will not only contribute to a better understanding of the virus biology, but may also facilitate the design of effective inhibitors to block virus entry.

## Introduction

Viruses are obligate intracellular parasites requiring access to the intracellular environment in order to replicate. Binding of the virus particle to the plasma membrane of the target cell is an important first step in the viral replication process. It is mediated by specific receptors and attachment factors [1]. While attachment factors are only involved in the attachment of the virus particles to the cell surface, cellular receptors promote the entry of the virus. Many viruses exploit several attachment factors, co-receptors and receptors that are typically engaged in series and/or in parallel via complex kinetic pathways. It is experimentally demanding to demonstrate true receptor status and thus, binding entities have often been reconsidered from being attachment factors to being receptors, as their roles in entry have become clarified. Some viral receptors have endocytic functions, such as transferrin and low-density lipoprotein receptors, while others exhibit alternative transport functions or are involved in binding to the extracellular matrix [2], illustrating how a variety of recognition events can be hijacked by viruses when entering cells. Even though the attachment factors are not directly involved in the entry of the virus into the cell, they are important for initiation of infection [1]. They have been suggested to concentrate and orient the virus near the cell surface and to allow the virus particles to get in close contact with their membrane-bound receptors, adding to the molecular and spatiotemporal complexity of the process. The presence of attachment factors and receptors is also a major determinant of the tropism of viruses, i.e., the selective ability to infect only specific species, tissues, and cell types [1]. The tropism of a virus thus determines the pathogenesis of the disease and defines the mechanisms of transmission [3].

Although the virus uptake mechanisms are principally similar across viral species, the molecular details are specific for each virus/target cell combination. Viruses are not passive cargoes, but trigger internalization by activating endocytic processes. This can be done by activating signaling pathways through direct or indirect contacts with cell-surface molecules and structures, including triggering of clathrin coat formation [3]. Different endocytic mechanisms including clathrin-dependent and clathrin-independent endocytosis and macropinocytosis have been reported for viral entry [2]. Moreover, even for one type of virus, there may be multiple pathways leading to productive viral replication. In addition to binding and entry, the passage of the virus particle, or at least of the viral genome, through a membrane into the cytosolic environment is a critical step in viral entry. This process is called penetration and often occurs in the endosome (endosomal escape). While non-enveloped viruses penetrate the endosomal membrane by membrane lysis or by pore formation, enveloped viruses fuse their membrane with the cellular membrane [3]. The penetration is activated by cellular cues, for instance a pH decrease through the endosomal system. Fusion of enveloped viruses is typically primed as a conformational change of a fusion protein in a process which frees a hydrophobic part of the protein, the fusion peptide. This is followed by insertion of the fusion peptide into the host cell membrane pulling the viral membrane and cellular membrane closer together [4–6]. This leads first to hemifusion, i.e., lipid exchange between the proximal membrane leaflets, followed by the complete fusion of both leaflets of the two membranes, resulting in the formation of a fusion pore and release of the viral genome into the cytosol.

It is worth noting that individual virus-receptor interactions at the cellular membrane are typically weak and provide only short residence times. Virus binding therefore usually involves the parallel engagement of many such interactions, thereby forming so-called multivalent interactions [7]. Such low affinity interactions are frequently mediated by glycoconjugates in the forms of glycoproteins and glycosphingolipids (GSLs), the glycan often being crucial for the interaction [8–10]. These glycoconjugates commonly function as receptors, but many viruses also take advantage of them as attachment factors, as is the case for negatively charged sialylated glycans or glycosaminoglycans such as heparan sulfate [11]. Such interactions can be primarily electrostatic in nature, and thus rather unspecific, but not seldomly also highly specific. Due to an exponential dependence between residence time and the strength of the interaction, the number of receptors bound by the virus (the valency of the interaction), has a much stronger than linear dependence on residence time of the virus, as manifested in non-trivial dependencies between receptor concentration and virus binding rate, also called superselectivity [7, 12, 13]. Multivalency is critical for appropriate attachment of viruses, both in terms of attachment strength and residence time, both of which are essential to commence cellular uptake or entry of the bound viruses. Furthermore, multivalency does not only prolong and strengthen the interaction between the virus particle and the host cell, but also provides the basis for mechanisms that are not available in monovalent systems [7]. For instance, multivalency provides opportunities to control the movement of virus particles on the cell surface as the lateral diffusion of a bound virus particle decreases with the number of interaction points. Importantly, multivalency also facilitates reorganization of the membrane. For example, the multivalent binding of GSL receptors has been shown to induce formation of membrane invaginations that are scissioned into endocytic vesicles transporting the virus to the endosome [14].

Due to the importance of virus-receptor interactions for viral entry, virologists, chemical biologists, biochemists, and biophysicists alike have all shown great interest in untangling the complex mechanisms governing receptor-mediated viral uptake. Such knowledge does not only contribute to a better understanding of infection biology, but may also facilitate the design of effective inhibitors to block viral entry pathway(s). Such insights are urgently needed not only to combat existing viral diseases, but also to rapidly react against emerging viral pathogens, such as the severe acute respiratory syndrome coronavirus 2 (SARS-CoV-2). This review focuses on selected key biomolecular processes mediating virus attachment and entry, all relying on the dynamic molecular interactions between viral proteins and cell-surface molecules. In particular, it highlights how recent methodological developments can contribute to a deeper understanding of such dynamic interactions. It further illustrates and discusses how such knowledge can contribute to the development of antiviral drugs targeting viral adhesion proteins, cellular receptors/attachment factors, their interactions, or factors essential in driving such interactions.

## Model viruses

To reflect the diversity of virus binding and entry as well as the need for different methodological approaches to study such processes, we have selected a few well-characterized non-enveloped (simian virus 40 and norovirus) and enveloped (influenza A virus, herpes simplex virus-1, and human immunodeficiency virus type 1) model viruses. Basic characteristics of these viruses are summarized below and in Table 1.

**Table 1:**
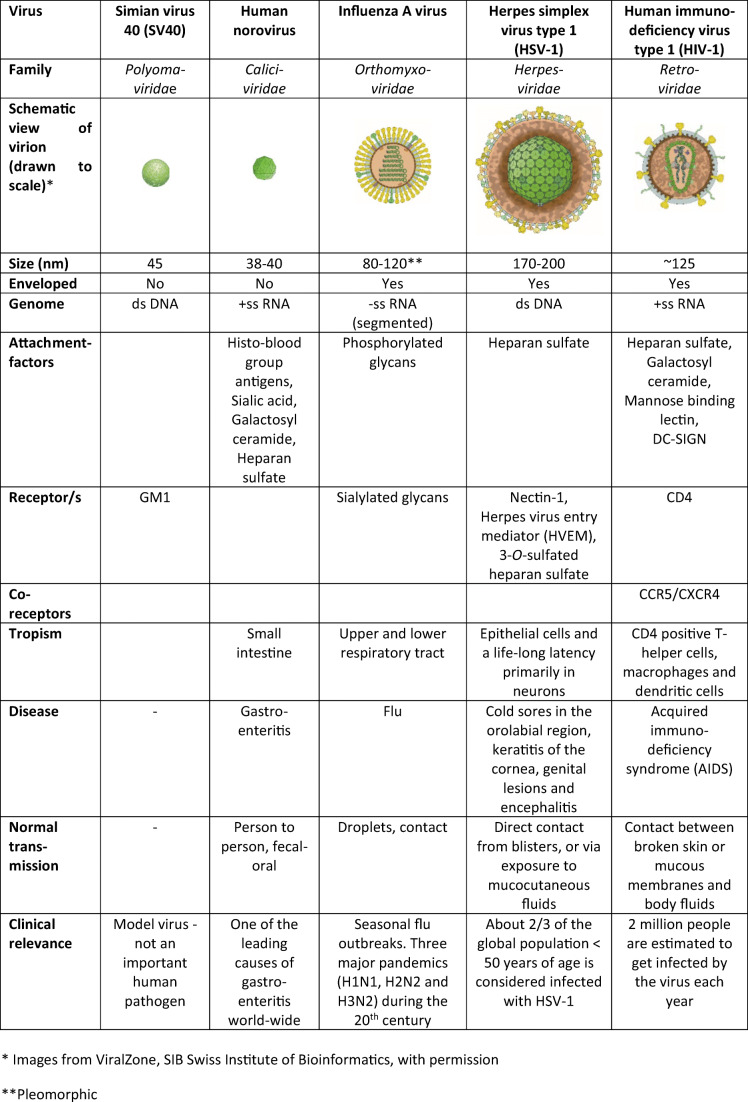
Characteristics of model viruses used to illustrate various concepts for virus-host interactions

### Simian virus 40

Simian virus 40 (SV40) belongs to the *Polyomaviridae* and is a non-enveloped DNA virus (Table 1). The SV40 capsid has a diameter of ~ 45 nm and its external surface is composed of 360 copies of the VP1 protein organized into pentamers. Each VP1 protein has one binding site for the α2,3-sialylated GSL GM1 [15], which is the cellular receptor for the virus [14, 16]. The affinity of the capsid is higher to the sialic acid variant Neu5Gc than to Neu5Ac [17]. Humans lack expression of Neu5Gc because of an inactivating mutation in the gene coding for the rate-limiting enzyme in Neu5Gc biosynthesis [18]. This is one of the reasons why SV40 infects human cells less efficiently than cells from monkeys, the natural host of the virus [17], and accordingly, the virus is not an important human pathogen [19]. Nevertheless, it is included in this review since it has been widely studied as a model virus thereby contributing to the understanding of viral entry and viral DNA synthesis.

### Norovirus

Norovirus is a non-enveloped positive-sense single-stranded RNA virus (Table 1). It belongs to the *Caliciviridae* and is divided into ten genogroups (G), of which GI, GII, and GIV infect humans [20]. The virus has a diameter of ~ 40 nm and is made up of an icosahedral capsid built of 90 dimers of the major capsid protein VP1 [21]. Despite intense efforts, the receptor of the human norovirus remains unknown, but susceptibility to infection requires expression of specific ABO(H) and Lewis histo-blood group antigens (HBGAs), which are recognized by the virus in a strain-dependent manner [22]. Most strains bind to the α1,2-fucosylated glycans of the HBGA family and individuals of the so-called non-secretor type, which lack expression of these glycans on the mucosal epithelia, have been shown to be resistant to such strains. The HBGAs are found both on glycoproteins and on glycosphingolipids [23]. In addition, human norovirus has been shown to bind to heparan sulfate [24], to sialylated glycans including SLex [25] and gangliosides [26], as well as to the GSL galactosylceramide [27]. Norovirus is included in this review because of its binding to HBGAs, which has been thoroughly investigated in a number of lipid-membrane-based assays. The fact that the virus binds to several structurally closely related glycans has allowed for interesting comparisons that have unraveled the detailed characteristics of virus attachment and detachment to membrane-bound GSLs. Finally, the interaction with HBGAs has been shown to be critical for norovirus infection as the expression of these glycans determines susceptibility to the infection [22, 23].

### Influenza A virus

Influenza A virus belongs to the *Orthomyxoviridae* family and is a negative-sense RNA virus with the genome divided into eight segments (Table 1). The virus particle is enveloped and has a cross sectional diameter of ~ 100 nm. The length of the particle, however, may differ substantially from this number, when appearing in spherical, elliptical and even filamentous shapes, depending on the IAV strain. The receptor of the virus was early suggested to contain sialic acid, but despite intensive studies, it is still not known whether the receptor is found on a glycoprotein or on a GSL [28]. Interestingly, strains of human influenza A virus prefer to bind to sialic acid with an α2,6-linkage to galactose while strains of avian influenza A prefer α2,3-linked sialic acid [9, 28, 29], which limits the spread between species. The binding to sialic acid is mainly mediated by the hemagglutinin (H_n_) protein, which in addition is also responsible for the pH-induced fusion of the viral envelope with the endosomal membrane of the target cell. In addition to the hemagglutinin, the viral membrane also contains a neuraminidase (N_n_), which binds to, and cleaves off, sialic acids from the cellular receptors. The balance of these two viral activities is considered critical for the whole infectious process [30, 31]. Recently, it was found that several influenza A viruses also bind to phosphorylated, non sialylated *N*-glycans, present in the human lung, which may explain why desialylation with neuraminidase does not block infection [32]. Influenza A virus is included in this review because of its well-characterized interaction with sialylated glycans, which has been studied with a large array of bio(physico)chemical approaches ranging from ensemble averaging methods, to high-resolution optical microscopy to atomic force spectroscopy. Such investigations of this virus led to the first demonstration that off-rates (desorption or unbinding rate) can be extracted from studies of single virus particle binding to membranes.

### Herpes simplex virus type 1

Herpes simplex virus type 1 (HSV-1) is an enveloped DNA virus belonging to the *Herpesviridae* family (Table 1) [33, 34]. The particle has a diameter of 170–200 nm [34] and contains 15 proteins, of which the 4 glycoproteins gD, gH, gL, and gB are considered necessary for cell entry [35]. HSV-1 uses heparan sulfate as the attachment factor and the interaction is mediated by gB together with gC. The three host factors nectin-1, herpes virus entry mediator (HVEM), and 3-O-sulfated-heparan sulfate can function as receptors for the virus, either alone or in combination. The binding of one of these receptors triggers a conformational change in gD, which thereafter interacts with the viral heterodimer of gH/gL. This interaction in turn causes an activation of gB, which is the protein that mediates the fusion with the cell membrane. The virus seems to enter different types of cells by different mechanisms [35]; it has been suggested that it enters epithelial cells by endocytosis and neural cells by fusion at the plasma membrane [35]. HSV-1 is included in this review as an example of a glycosaminoglycan-binding virus. Importantly, studies of HSV-1 have shown that the strength of the binding to glycosaminoglycans determines the diffusion of the virus particle on the plasma membrane and that proteoglycans have the potential to be functional receptors.

### Human immunodeficiency virus type 1

Human immunodeficiency virus type 1 (HIV-1) belongs to the *Retroviridae* family (Table 1). The virus particle is enveloped and has a diameter of ~ 125 nm [36]. HIV-1 has a positive single-stranded RNA genome that is converted to DNA in infected cells by the viral reverse transcriptase before being integrated into the host genome. HIV-1 primarily infects immune cells which express the viral receptors and the primary receptor of the virus is the cluster of differentiation 4 (CD4) glycoprotein. The interaction with this receptor leads to exposure of binding sites allowing for interactions with chemokine receptors, i.e., CCR5 and/or CXCR4, which are co-receptors for the virus. The viruses that mediate primary infections typically use CCR5 as co-receptor, while viruses associated with late-stage infections usually use CXCR4 [36]. Heparan sulfate, galactosylceramide, mannose-binding lectin, and DC-SIGN have all been identified as attachment factors for the virus [37]. The entry of the virus particle into the cell is usually considered to be mediated by fusion at the plasma membrane, but fusion can also occur after endocytosis of the virions [36]. HIV-1 is included in this review as an example of a virus using protein receptors and because biophysical investigations have uniquely contributed to providing mechanistic details of the fusion process.

## Methodological considerations

As virus-receptor interactions are of paramount importance for virus attachment to target cell membranes and often drive virus internalization, their characteristics have been the subject of intense research. A multitude of methods have been applied in such studies, probing the interactions on different levels of complexity (e.g., either employing the entire, membrane-associated receptor complex, utilizing isolated ligand-receptor pairs, or even focusing on specific protein domains such as receptor binding domains) and providing quantitative insights into the interactions. For example, isothermal titration calorimetry (ITC) has been used to determine the heat released by binding of attachment factors to viral proteins, yielding information on dissociation constant *K*_d_, the change in free enthalpy (Δ*H*), and free energy (Δ*G*) associated with the binding process [38, 39]. In these experiments, isolated virus proteins and attachment factors are typically used, yielding information on the monomeric interaction. Nuclear magnetic resonance (NMR) spectroscopy measures changes of chemical shifts of residues involved in the binding process, thus yielding the engagement of the viral proteins versus increasing concentrations of attachment factors [40, 41]. These binding curves typically possess a sigmoidal shape if plotted as function of the logarithm of the attachment factor concentration. The inflection point of this curve corresponds to the *K*_d_ value of the interaction, which is typically determined by non-linear least squares fitting of a Langmuir-type binding isotherm to the data. Such experiments have been done using both isolated viral proteins and entire viruses, but are typically limited to the use of truncated variants of the membrane receptor.

**Fig. 1:**
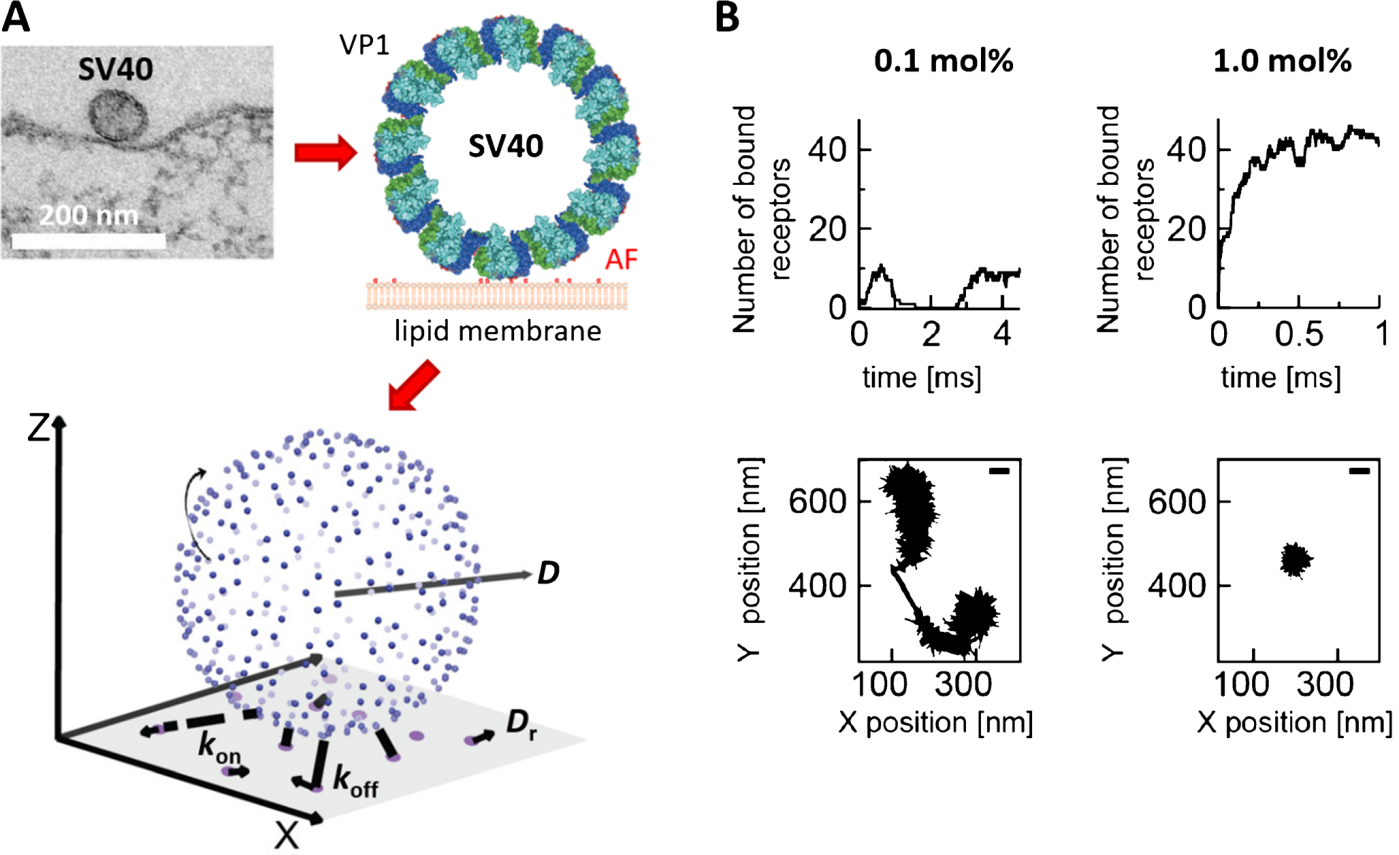
Virus binding to cell membranes is a complex and highly dynamic process. (A) Viruses are known to present multiple copies of proteins (e.g., VP1 in the polyomavirus SV40) that are able to bind to receptors or attachment factors (AFs) presented by the cell’s plasma membrane. In order to achieve firm attachment to cell membranes, viruses typically have to bind many receptors/attachment factors in parallel, which leads to a very dynamic and complex binding process. The understanding of this process requires precise knowledge on the properties of the constituents (e.g., the mobility *D* of receptors/attachment factors in the membrane, the rate constants *k*_on_ and *k*_off_ of the molecular interaction between single viral proteins and their receptors/attachment factors) and the underlying biophysical laws, which can be obtained using biochemical and biophysical techniques (some of which are discussed in this review), but often requires to approximate the native situation by simplified model systems (e.g., by approximating virus-cell interactions by the interaction of virus-like particles with less complex lipid bilayers). Such investigations have, for example, enabled to generate a numerical model of SV40 that allows to study the impact of receptor concentration, mobility, and binding strength (A, lower panel) on the dynamics of virus attachment and the motion of membrane-bound viruses (B). Adapted with permission from [42]

**Fig. 2:**
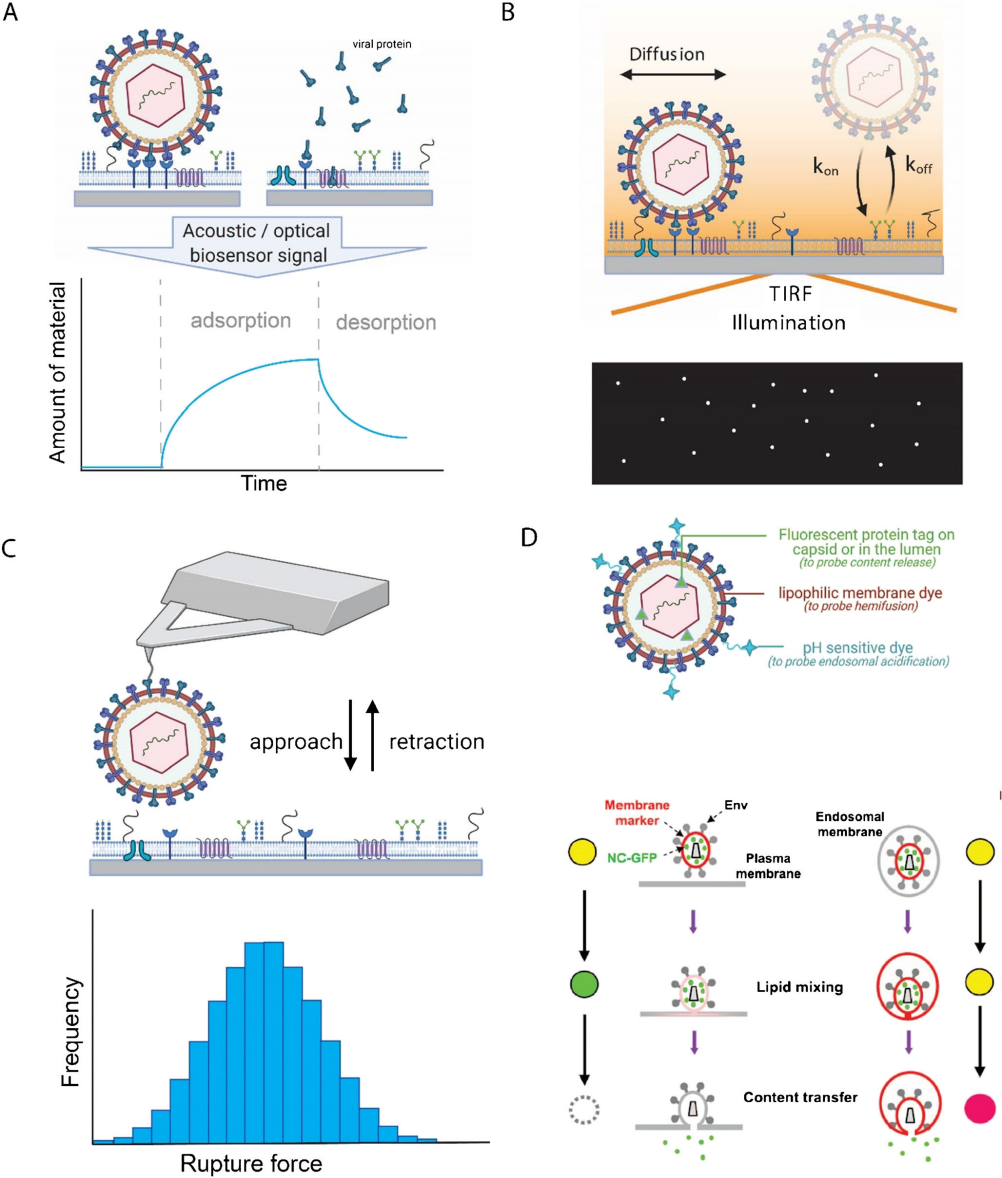
Bioanalytical methods to probe virus-membrane interactions. (A) A surface-based biosensor monitors in real time the change of an acoustic or optical signal upon adsorption of a virus particle or viral protein to surface-deposited receptors or cell surface mimics. In such an experiment, one of the binding partners is immobilized at the surface of a sensor chip while the other binding partner is added using a flow chamber. The acoustic or optical signal generated upon binding at the biosensing interface can be related to the amount of material deposited at the sensor surface. Analysis of the signal upon virus or protein adsorption together with analysis of the detachment behavior upon rinsing yield information on the ensemble-averaged interaction kinetics and affinity. (B) Binding of single viruses to membranes can be efficiently probed using total internal reflection fluorescence (TIRF) microscopy, which generates a very short-ranged evanescent excitation field at the interface, making it possible to visualize surface-bound fluorescently labeled viruses while discriminating them from the ones in solution. Analysis of the rate of arrival under equilibrium conditions yields information on the association behavior of the virus particles. Analysis of the residence time, i.e., the time spent at the surface before detachment yields information about the dissociation rate constant (k_off_). Additionally, TIRF microscopy allows for single particle tracking (SPT) which can be used to characterize the diffusive behavior of the surface-bound virus particles. (C) Atomic force microscopy (AFM)-based dynamic force spectroscopy (DFS) is used to probe the rupture forces between a virus particle bound to a sharp AFM tip and sensor-immobilized cellular ligands, yielding histograms of the rupture forces. Analysis of the distributions of detachment forces at different loading rates (dynamic force spectroscopy; DFS) yields information on the energy landscape of the interaction and on the equilibrium off-rate. (D) Labeling strategies to probe the viral fusion process. The virus envelope can be tagged with a membrane-inserting dye or a fluorescent lipid to probe hemifusion/lipid mixing. A pH-sensitive tag can further be added to the particle surface to monitor the changes in pH experienced by the virus. A fluorescent protein can be attached through genetical engineering to the capsid, or to an accessory protein within the virus lumen to visualize content/capsid release into the cytosol. Adapted with permission from [45]

In principle, methods such as ITC and NMR can be used to determine the monovalent binding affinity of viral proteins interacting with water-soluble attachment factors. The overall (multivalent) interaction of a virus with a cell membrane shows, however, a very complex dependence on multiple parameters (Fig. 1), such as the monovalent binding affinity, the valency, and the mobility of the receptors in the membrane, and can, in principle, be further modified by steric hindrance or (anti-)cooperativity effects [43, 44], causing the (monovalent) binding affinity in a multivalent complex to decrease or increase depending on the number of interactions engaged. Quantifying the overall interaction avidity therefore requires approaches able to probe the interaction of viruses with receptors or attachment factors in a biomimetic environment, e.g., by embedding host membrane components in lipid membranes (e.g., lipid-bound factors) or attaching them at interfaces (e.g., for glycosaminoglycans). Such measurements can be conducted using surface-sensitive techniques such as quartz crystal microbalance with dissipation (QCM-D), surface plasmon resonance (SPR) spectrometry, or biolayer interferometry (Fig. 2A). QCM-D is an acoustic biosensing technology that allows to monitor adsorption and desorption processes at interfaces such as the binding of viruses to receptor-presenting membranes; it provides information on surface coverage in terms of adsorbed mass per unit surface area as well as the hydrodynamic friction which represents the viscoelastic and structural properties of interfacial films [46, 47]. However, the probed mass also includes hydrodynamically coupled liquid, which makes absolute quantification of mass coverage somewhat uncertain, although relative variations can be quantified with high accuracy [47, 48]. SPR and biolayer interferometry are label-free optical biosensing technologies that make it possible to follow, in real time, the adsorption of a ligand to a receptor immobilized on the sensor by following changes in the refractive index occurring at the interface. In contrast to QCM, the change in the refractive index is measured with references to the refractive index of the surrounding solution, which makes it possible to quantify the biomolecular coverage given that the refractive index of the biomolecule is known [49, 50]. By analyzing the amount of ligand immobilized at the surface as a function of the ligand concentration in solution, or by following the adsorption and desorption kinetics at varying protein concentrations, the affinity (*K*_d_) is obtained. The dissociation rate constant (*k*_off_), a measure of the bond stability, can be quantified by recording protein detachment upon rinsing. These methods are compatible with the use of receptor/attachment factor-containing supported lipid bilayer (SLB) at an interface for real-time measurements of the binding kinetics of viruses as function of virus concentration [51, 52]. Such data allows to extract a *K*_d_ value of the overall interaction (avidity) as well as on- and off-rates (*k*_on_ and *k*_off_) by modelling the data using dedicated binding models. However, due to the often very long residence times associated with multivalent interactions and the notable heterogeneity within virus populations (e.g., for pleomorphic viruses), it is generally very difficult to relate such ensemble-averaged data (i.e., a response which is averaged over all viruses probed) to molecular properties of the monovalent interactions (affinity). This challenge can be efficiently tackled using methods that probe interaction kinetics at the single particle level. In contrast to ensemble averaging techniques, single particle techniques are sensitive enough to extract statistics from multiple monovalent binding events, simultaneously also providing information about binding heterogeneity originating from multivalent interactions.

An experimental setup to study the binding of virus particles to cell-surface components on a single particle level takes advantage of total internal reflection fluorescence (TIRF) microscopy (Fig. 2B). In this imaging method, the solute-surface interface is illuminated using an excitation beam that hits the interface at the critical angle of total internal reflection, creating an evanescent excitation field, the intensity of which decays exponentially with the distance from the interface [53]. As typical decay lengths are in the order of 100 nm, only fluorescently labeled objects in direct vicinity to the interface are excited and contribute to the image generation, while particles in solution are not excited and thus not imaged by the microscope. Using this imaging approach, the binding and release of individual particles can be monitored under binding equilibrium conditions, yielding quantitative information on *k*_on_, *k*_off_, and hence *K*_d_ thanks to a data analysis procedure referred to as equilibrium fluctuation analysis (EFA) [54, 55]. Additionally, single particle tracking (SPT) can be used to gain information on the diffusive behavior of the receptor-bound virus particles.

Furthermore, molecular details of the detachment behavior of individual receptor-bound virus particles can be obtained using dynamic force spectroscopy (DFS). For example, in DFS experiments using atomic force microscopy (AFM), a virus particle is immobilized at the end of a sharp AFM tip and approached to a receptor-presenting surface (Fig. 2C). The tip is then retracted, thereby applying a controlled external force which is increased over time until unbinding (bond rupture) is observed [56]. Thus, DFS allows, in contrast to conventional optical microscopy, to load the virus-receptor interaction with a well-defined external force and therefore provides information on the stability of the interaction in dependence of the applied force load. Interestingly, such a detachment process (and bond failure in general) is a stochastic process, which means that the observed detachment force often shows a notable variation even when keeping the experimental conditions fixed [57]. Theoretical assessments of DFS experiments have shown that the mode of the corresponding detachment force distribution (i.e., the value of the most frequently observed detachment force) increases with increasing loading rate (i.e., the rate of force increase), which allows to gain information on the shape of the interaction energy landscape, such as the change in free energy associated with unbinding, the width of the binding potential, as well as the zero-force off-rate (determined by extrapolating the unbinding dynamics towards zero force) [57].

In addition to the procedures described above to probe virus-membrane interactions on a single particle level, a number of assays have been designed to specifically investigate the different steps leading to virus entry by fusion, and release of the viral content/genome, on a single particle level. In all cases, such assays rely heavily on the use of virus particles labeled with multiple fluorophores. Often, a lipophilic membrane dye is included within the viral lipid membrane at self-quenching concentrations [45, 58, 59], allowing for the observation of lipid mixing and of the hemifusion and fusion process (Fig. 2D) [45, 58, 59]. In addition to this, a soluble fluorescent marker can be included in the lumen of the viral particle [45, 58, 60], or the viral capsid can be labeled directly [60–63]. Both strategies allow for the visualization of pore formation and viral content release (Fig. 2D). Finally, a pH-sensitive dye may be added to the virus particle surface, to follow endosomal acidification and thus correlate the fusion steps with ambient pH [60–62]. As further exemplified for the HIV-1 fusion process, this multi-label strategy can be used in combinations with cell membrane mimics (in the form of liposomes [64], giant unilamellar vesicles [63, 65], or supported lipid bilayers [58, 63, 65–68]) as well as with live cells [5, 45, 69–72].

### SV40 and norovirus as models to study the binding of non-enveloped viruses to glycans

The kinetic analyses of virus binding and release are simplified if the viruses under investigation share the same well-defined geometry. This condition is fulfilled for many non-enveloped viruses, which are made of a highly regular capsid and whose capsid proteins can adopt only a single or very few geometrical configurations [73, 74]. Accordingly, first attempts to acquire molecular information from the measurement of virus-membrane interactions were done using either the polyomavirus SV40 or human noroviruses. For example, Szklarczyk et al. [42] provided a detailed picture of the interaction of SV40 with its receptor, the GSL GM1, by feeding data obtained from experiments using GM1-containing supported lipid bilayers (SLBs) into a computational model of the SV40-GM1 interaction (Fig. 1). In this case, the authors combined information from QCM-D and fluorescence microscopy measurements to gain knowledge about the diffusion of the GM1 GSL within the lipid membrane and the binding of SV40 to the GM1-containing SLBs, respectively. Interestingly, the QCM-D measurements revealed that the dependence of the SV40 surface coverage on the receptor concentration is highly non-linear, starting with negligible SV40 binding at low GM1 concentrations (< 0.15 mol %), followed by a steep increase in the SV40 surface coverage and a saturation of the SV40 coverage after exceeding a threshold concentration; a similar dependency has also been observed for norovirus [51]. Using these experimental data, the authors were able to refine their computational model of the SV40-GM1 interactions and to provide simulations on the time-dependent engagement of GM1-binding sites on the SV40 capsid, as well as estimates of the valency of the membrane-bound SV40, i.e., number of SV40-GM1 links, in dependence of the receptor density in the planar membrane (Fig. 1). The analysis suggested that the SV40 virus needs to bind at least 4 GM1 lipid molecules to achieve firm/irreversible attachment to the SLBs, thereby providing a molecular picture for the strong non-linearity observed in the SV40 surface coverage.

**Fig. 3:**
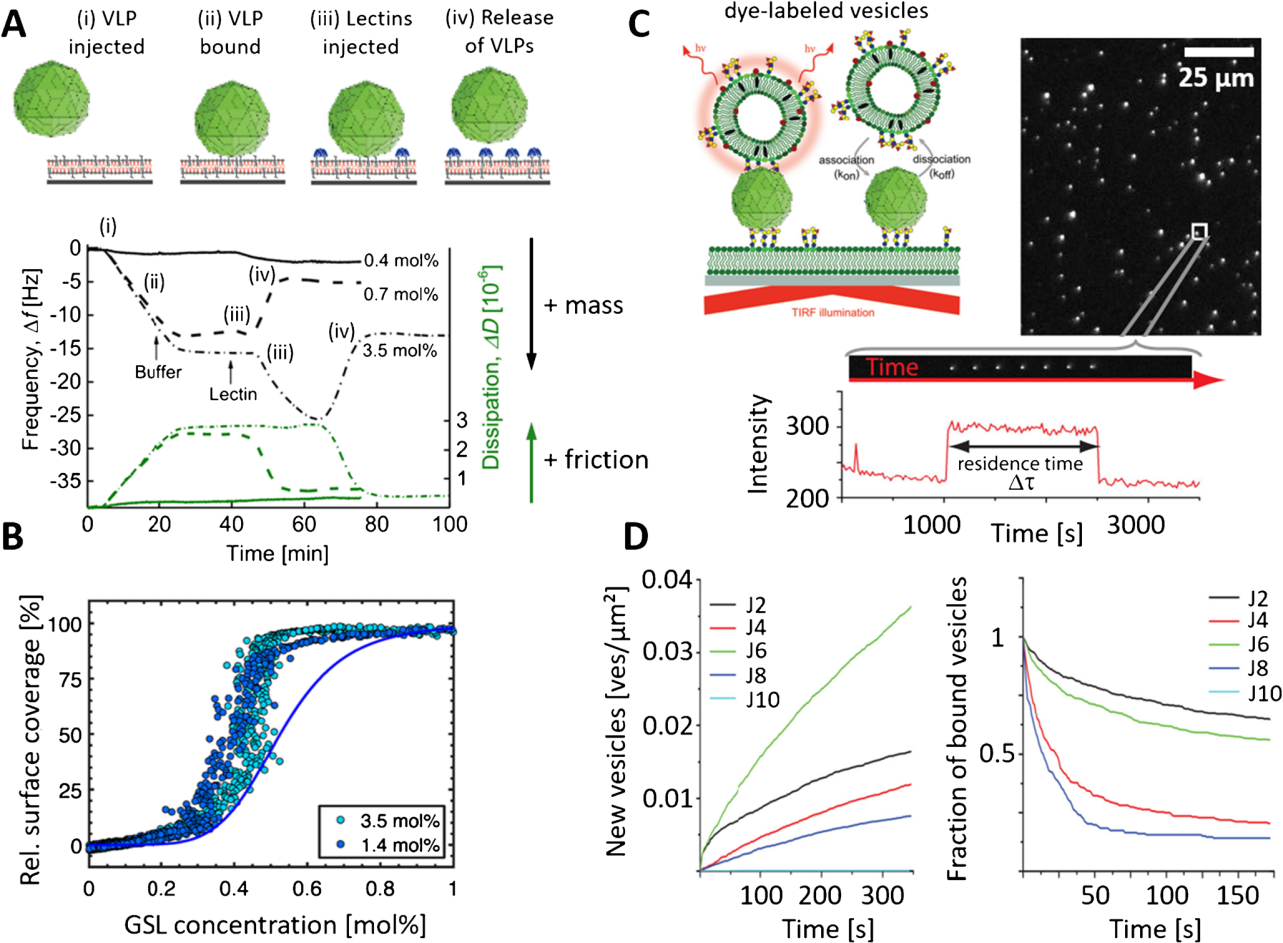
Probing the binding of norovirus virus-like particles (VLPs) to membranes using a quartz crystal microbalance with dissipation monitoring (QCM-D; A, B) and total internal reflection fluorescence (TIRF; C, D) microscopy. (A) QCM-D allows to probe the binding of viruses or lectins to receptor/attachment factor-presenting membranes by providing information on the adsorbed mass (measured in terms of a resonance frequency *f*, the value of which decreases with increasing mass) and the interfacial hydrodynamic friction (measured in terms of a dissipation *D*, which increases with increasing friction). As the friction strongly depends on the size of the adsorbed object, it is possible to distinguish binding events caused by viruses and proteins and thus to probe the release of membrane-bound viruses upon addition of a binding competitor having a higher affinity to the receptor/attachment factor (e.g., a lectin). This provides information on the binding strength of the virus-membrane interaction and (B) on the accumulation of receptors/attachment factors by the virus, which generates deviations between the relative virus surface coverage, determined during the initial binding process (B, solid line) or lectin-induced virus release (B, circles) [78]. (C) TIRF microscopy can be used to probe the binding of receptor/attachment factor-containing, dye-labeled vesicles and interface-linked viruses, allowing to determine the impact of the constituents (e.g., vesicle composition or virus strain) on the overall interaction. (D) This approach was used to probe the interaction of individual norovirus particles to lipid membranes obtained from lipids extracted from human intestinal enteroids established from various individuals. Thanks to such an experiment, the attachment rates (slopes in D, left) as well as the off-rates (decay of residence time, D right) of virus-membrane interactions can be related to the patterns of susceptibility to infection for various individuals [23, 55]. Adapted figures with permission from [23, 55, 78]; Copyright by the American Chemical Society and American Physical Society, respectively

Additionally, the QCM-D measurements showed that once SV40 bound to a GM1-containing SLB, the viruses remain bound even after an extensive buffer rinse. Hence, the interaction appears to be basically irreversible in the absence of external forces and accordingly, such QCM-D measurements provide only information on the rate of SV40 attachment but not on the detachment. In order to tackle this limitation, Parveen et al. [75] investigated the possibility to induce SV40 detachment by addition of a GM1 binding competitor to SLB-bound SV40. Their study relies on cholera toxin subunit B (CTxB) which possesses a much higher affinity to GM1 in comparison to SV40’s VP1 (*K*_d_ << 0.1 μM vs. ~ 5 mM, respectively) [15, 76, 77], and can accordingly induce release of membrane-bound SV40. As SV40 and CTxB differ strongly in their liquid-related hydrodynamic friction and thus generate different dissipation traces in QCM-D measurements, it was possible to determine the individual contributions of SV40 and CTxB to the measured interface-bound mass. This, in turn, made it possible to probe the release kinetics of SV40 in dependence of the binding dynamics of CTxB. These measurements showed that a substantial amount of GM1 receptors had to be engaged by CTxB (> 90%) before SV40 release was triggered, indicating that CTxB is able to bind to GM1 that were formerly bound to SV40 and thus that depletion of GM1 (caused by engagement with CTxB) reduced the valency of membrane-bound SV40 until release was induced. By solving the rate equations of CTxB binding and SV40 release and by using estimates for the maximum valency obtainable by SV40 on a planar membrane, it was possible to relate the observed release kinetics with the binding strength of the single VP1-GM1 pair. In a follow-up study, Parveen et al. [78] applied the same methodology to probe binding of human norovirus VLPs to histo-blood group antigens (HBGAs) presented on GSLs and release of these VLPs from the HBGAs upon addition of a lectin from *Ralstonia solanacearum* (Fig. 3A, B). The norovirus and the lectin bind with different affinities to two HBGA GSLs, i.e., H type 1 and B type 1, and thus allow to compare the impact of their different binding affinities to the norovirus release dynamics. In comparison to the competitive release of SV40, norovirus release was induced at much larger relative surface coverages of either of the HBGA GSLs (Fig. 3A). In addition, the data allowed to resolve that the lectin surface coverage at which the onset of release occurred, increased with increasing HBGA GSL concentration in the SLB. This behavior made it possible to use the lectin surface coverage as an indicator for the amount of binding-competent HBGAs and thus to probe norovirus release upon subsequent reduction of the HBGA concentration (due to engagement with the lectin). A comparison of the norovirus attachment kinetics (measured for SLBs containing different amounts of HBGA GSLs) with the so-obtained detachment kinetics (Fig. 3B) showed that both dynamics were in good agreement for binding to H type 1. However, similar comparison revealed a significant deviation for norovirus binding to B type 1, which indicated that the norovirus to B type 1 interaction is strong enough to increase the HBGA GSL concentration within the virus-membrane contact area (i.e., to induce a notable HBGA GSL accumulation with respect to the remaining SLB).

These investigations indicate that receptors/attachment factors engaged in virus binding can participate in a dynamic equilibrium with non-engagedreceptors/attachment factors embedded in lipid membranes. The results also provided estimates for the strength of monovalent interactions between the viral binding protein (VP1) and receptors/attachment factors (HBGA) embedded in a lipid membrane. This estimate, however, relied on assumptions on the maximum valency achievable by the virus on a planar membrane, which was derived in these studies from geometrical considerations. In order to relate the observed virus attachment and release rates with molecular reaction rates, knowledge about the valency distribution of the viruses probed is needed, which requires methods to probe the binding and release of individual virus particles. Such an approach was introduced by Bally et al. [55], who linked VLPs of a norovirus GII.4 strain to an interface and monitored the binding of single HBGA GSL containing, fluorescently labeled, liposomes to the viruses using TIRF microscopy (Fig. 3C). This approach yielded information [55] on the binding rate of liposome attachment to the norovirus VLPs as well as the distribution of the liposome residence time on the VLPs using the EFA data analysis method (Fig. 3C, D). The single virus particle resolution provided by TIRF microscopy imaging allowed the identification of subtle variations in the binding to H type 1 and Lewis b HBGAs, such differences that were hidden in conventional ensemble-averaged measurements. Furthermore, information on the rate of bond breakage (off-rate, *k*_off_) should be extractable from the measured residence time distributions, which was demonstrated in interaction studies of fixed valency. The corresponding distributions obtained from the norovirus-HBGA interactions were, however, too broad to allow for the extraction of molecular off-rates, which was attributed to a notable variation of the binding valency within the ensemble (caused by a notable variation in size of the liposomes and possibly also in their HBGA content). A similar experimental approach was used recently by Rimkute et al. [23] to probe the interaction kinetics between another GII.4 norovirus VLP and HBGA-containing vesicles, produced from lipids extracted from human intestinal enteroids. Here, the enteroids were established from biopsies of six individuals exhibiting distinct histo-blood group antigen profiles and varied in susceptibility to norovirus infection (Fig. 3D). This experimental approach made it possible to relate virus-membrane interaction kinetics to susceptibility patterns and to pinpoint kinetic features which may be of relevance in this context.

Studies of fluorescently labeled norovirus VLPs have revealed that the VLPs cluster on supported lipid bilayers containing GSLs [79]. Larger clusters were observed on bilayers containing GSLs with a higher affinity for the VLP. It was hypothesized that the clustering was promoted by virus-induced membrane deformation. Indeed, norovirus VLPs have been shown to induce membrane invaginations upon binding to GSL incorporated into giant unilamellar vesicles [80] in a similar manner as SV40 [14]. For the latter virus, it has been proposed that such invaginations lead to the formation of vesicles that transport the viruses through the cytoplasm.

As the valency has a strong impact on the virus residence time [7], information on the valency and the residence time of the viruses is needed to extract the (valency-dependent) off-rate. Such information can, in principle, be obtained if the virus binds to receptors/attachment factors which are able to diffuse within a (fluid-phase) SLB. Indeed, complexation of SLB-embedded structures (e.g., lipids or membrane proteins) has been shown theoretically and experimentally, to reduce the diffusion coefficient of the complexes in the lipid bilayer [42, 81, 82]. In general, the diffusion coefficient of the complex (virus bound on a planar lipid membrane) decreases monotonously with increasing number of ligand-receptor pairs [83]. This allows us, in principle, to gain information on the valency of a SLB-bound virus by determining the lateral diffusion coefficient of single virus particles. Application of SPT to SLB-bound SV40 indeed showed a transition from full immobilization (no measurable diffusion) to random motion and release of SV40 upon addition of CTxB. This observation indicates that receptor depletion continuously lowered the SV40 valency causing virus mobility and finally virus release [75]. Nevertheless, the interaction between SV40 and GM1, as well as norovirus and H type 1 and B type 1 GSLs, is so strong that these viruses remain essentially immobile without addition of a binding competitor [75, 79], which has so far hindered the application of this methodology to extract the exact (valency-dependent)off-rate distributions.

### Influenza virus as a model for describing single particle interactions with sialic acids

The influenza A virus (IAV) which relies on sialic acids for attachment and entry into the host cell has been extensively used to initially demonstrate how off-rates of single viruses interacting with membranes can be extracted [84, 85]. Indeed, due to its huge relevance for the public health, IAV has become an important model virus in bio(physico)chemical investigations relying on methods such as SPR [86], NMR [40], X-ray diffractometry [87], biolayer interferometry [88], glycan arrays [89, 90], as well as various microscopic techniques [84, 85, 91]. In particular, both dynamic force spectroscopy (DFS) and optical microscopy have been used to probe virus-receptor interaction at different complexities in terms of the macromolecules involved in the experiment. Indeed, the interaction partners investigated with DFS range all the way from the use of truncated versions of viral proteins and receptors immobilized at defined interfaces [92], up to entire viruses interacting with the plasma membranes of host cells [84].

**Fig. 4:**
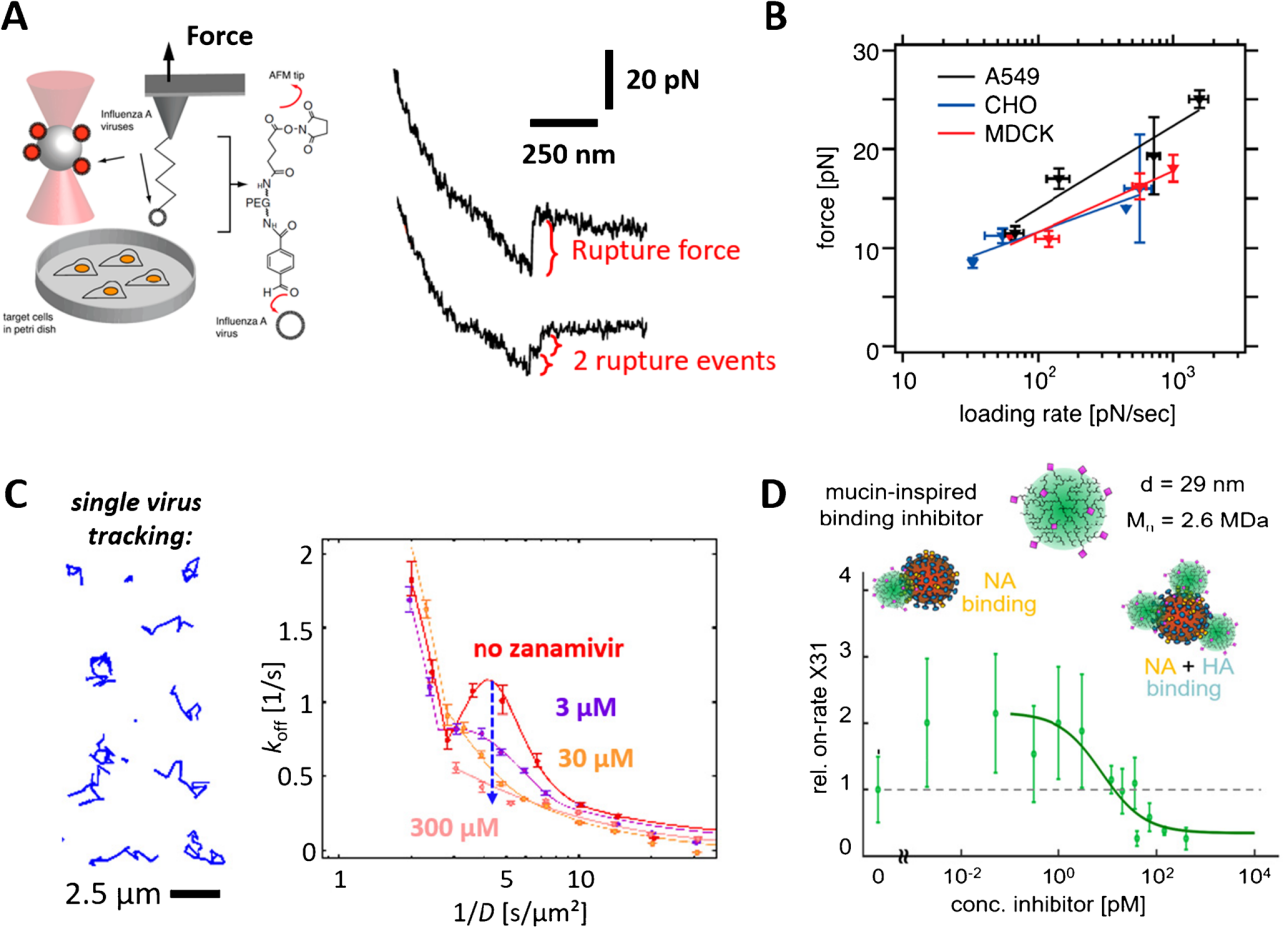
Probing the interaction of influenza A viruses (IAVs) with cells and attachment factors using dynamic force spectroscopy (DFS; A, B) and TIRF microscopy (C, D). (A) AFM-based DFS applied to the interaction of IAVs with cells showed mostly single rupture events, which are attributed to the unbinding of single viral proteins from sialylated structures on the cell’s plasma membrane. (B) The loading rate–dependent unbinding force showed only little variation across the different IAV strains and cell lines used, which is in line with previous studies showing only minor differences in the affinity to IAV’s protein hemagglutinin to α2,3- and α2,6-linked sialic acids [84]. Adapted figure with permission from (Sieben 2012); Copyright by the National Academy of Sciences. (C) TIRF microscopy applied to dye-labeled IAVs interacting with sialic acid–presenting supported lipid bilayers (SLBs) showed that due to the fluid nature of the membrane, IAVs can move laterally while being bound to the membrane (C, left). The speed of this process (quantified in terms of a diffusion coefficient) contains information on the number of attachment factors engaged by the IAV, which can be used to determine the relationship between IAV residence time and number of bound attachment factors and to monitor how this relationship is modified by neuraminidase inhibitors (C, right). Furthermore, by monitoring the frequency of virus binding to the interface, the assay also provides information on the rate of virus attachment (D). Addition of substances that inhibit virus binding (by either blocking the viral proteins or the attachment factors) causes the virus attachment rate to decrease with increasing inhibitor concentrations, which can be quantified using the assay and used to assess the performance of virus binding inhibitors [85, 93]. Reprinted figures with permission from [85, 93]; Copyright by the American Chemical Society and Wiley-VCH, respectively

The first demonstration of probing the binding strength of intact influenza viruses by force spectroscopy was done by Sieben et al. [84], who employed optical tweezers and AFM-based DFS to determine the force needed to detach IAVs (strains X31 and WSN/32) [56, 84] from the surface of live host cells [57, 84] (Fig. 4A, B). Single unbinding events, which showed detachment forces in the order of 10–20 pN (for loading rates ranging between 10 and 1000 pN/s) were observed most of the time (Fig. 4A) and were attributed to unbinding of single IAV hemagglutinins from sialylated structures presented on the cell surfaces. Surprisingly, only little variation in unbinding was observed using the different IAV strains and three different cell lines (Fig. 4B). As the virus strains used are known to differ in their binding preference to sialic acids (α2,3-linkage for WSN/32 and α2,6-linkage for X31) and as MDCK and A549 cells clearly express sialic acids with α2,6-linkage but CHO cells present only sialic acids with α2,3-linkages [89], this finding suggests that, on a molecular level, the hemagglutinins bind with comparable affinity to sialic acids in both α2,3- and in α2,6-linkages. This observation is in agreement with NMR measurements of binding affinity as well as with glycan array experiments, showing that a multivalent presentation of attachment factors can strongly enhance the preference for a particular linkage [40, 94]. Recent measurements show that both hemagglutinins and neuraminidases of IAVs are able to bind to sialic acids with comparable binding strengths, suggesting that both surface proteins of IAVs are involved in the cell-surface attachment of the virus [92]. This observation indicates that the role of neuraminidase in the IAV binding and unbinding process ranges well beyond the promotion of IAV egress through sialic acid cleavage activity. Hence, gaining more insights into the complex interplay of hemagglutinins and neuraminidases during IAV binding and unbinding requires probing these processes at equilibrium. The first measurement of the IAV binding and unbinding at equilibrium was done by Lee et al. [91], who used TIRF microscopy to monitor the transient attachment of the IAV strain X31 to SLBs containing sialic acid–presenting lipids (analogous to Fig. 2B). Application of the EFA method made it possible to determine how the lipid structure, i.e., the presentation of sialic acids by the lipids, modified the rate of IAV attachment. Similar to previous studies using norovirus [55], the measurements yielded very broad residence time distributions, which did not allow to extract off-rates of the interaction. This problem was approached by Müller et al. [85], who took advantage of the fact that lipids within fluid-phase SLBs are able to move laterally with a diffusion coefficient for the 2D diffusion of the sialic acid–bound IAV (Fig. 4C) indicative of the number of lipids bound by the tracked IAV [83]. This characteristic was used [85] to extract valency-dependent residence time distributions and thus to determine the IAV off-rate in dependence of a measure for the average number of bound attachment factors (Fig. 4C). Interestingly, the off-rate showed the expected decrease for increasing valency (caused by the fact that a higher valency requires more single interactions to be broken), but also an unexpected peak behavior, demonstrating that the off-rate increased above the expected values for certain binding valences. Application of the neuraminidase inhibitor zanamivir caused this peak to disappear, showing that this peculiar feature in the valency-dependent off-rate is caused by a competition in the opposing activities of the hemagglutinin and the neuraminidase. In a related study [93], the same assay was used to probe the decrease in IAV attachment rate caused by interaction with sialic acid–presenting polymers (Fig. 4D). Such polymer-based virus binding inhibitors are designed to bind specifically to IAVs by the hemagglutinin-sialic acid interaction and are therefore expected to inhibit IAV binding to sialic acid–presenting(cell) membranes. Application of the TIRF-based binding assay revealed a complex dependence of inhibitor concentration on the IAV attachment rate, which showed a biphasic behavior instead of the expected monotonous decrease and was attributed to an interaction of the sialylated compound with both proteins, the hemagglutinin and the neuraminidase (Fig. 4D). Furthermore, the assay enabled to probe changes in the multivalent interaction caused by addition of the virus binding inhibitors, which can be used to assess their inhibition efficiency.

These examples show that both methods, DFS and equilibrium binding probed using TIRF microscopy, are very powerful approaches for characterizing complex interactions on a molecular level. Due to their single-virus/single-molecule resolution, both techniques are able to extract information and identify subpopulations, which are typically lost in the ensemble-averaging process of traditional binding assays. This high resolution requires, however, more sophisticated and time-consuming data analysis schemes, which represents a notable bottleneck in inhibitor screening applications. Hence, ensemble-averaging and single-molecule binding assays complement each other in this respect, as their combinations enable an initial screening of many compounds, followed by an investigation on the binding properties of the most successful candidates using single-molecule binding assays.

### Herpesviruses as a model of glycosaminoglycan-binding viruses

Together with sialic acid moieties, sulfated glycosaminoglycans (GAGs) are the most common attachment factors for viruses and accordingly, a large variety of human viruses take advantage of these carbohydrates for their initial recruitment to the surface of host cells [8], including the human papillomavirus, filovirus, flavivirus, coronavirus families, and herpesviruses HSV-1 and HSV-2 [95]. GAGs are linear polysaccharide molecules consisting of repeating disaccharide units. They are major constituents of the glycocalyx, extending up to several 100 nm above the plasma membrane [96]. While the molecular players involved in virus-GAG recognition are often known, little attention has been devoted to the dynamics of such interactions. Indeed, it remains essentially unknown how the interactions are modulated to facilitate viral infection and to confer optimal attachment, detachment, and diffusion behavior to the virus at the cell surface.

**Fig. 5:**
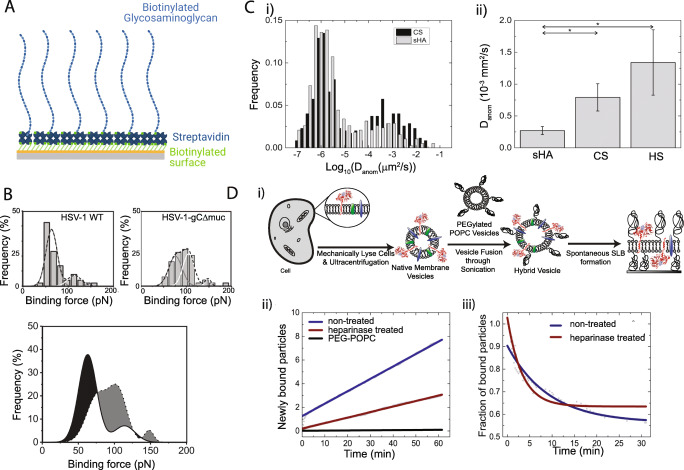
Probing HSV-glycosaminoglycan (GAG) interactions using surface-immobilized GAGs. (A) A biomimetic GAG-platform based on the end-on immobilization of GAGs via biotin-streptavidin is used to probe the interaction between GAGs and viral glycoproteins or viruses. (B) Dynamic force spectroscopy (DFS) experiments are used to probe the virus-GAG binding strength. The histograms, showing the rupture force frequency recorded between CS and HSV-1-WT, are fitted with multiple Gaussian curves for different ligand-receptor interactions (top) and then compared for HSV-1-WT and HSV-1-gC-Δmuc (bottom). Black-shaded areas show higher binding frequencies for HSV-1-WT, while the gray-shaded areas correspond to higher binding frequencies for HSV-1-gC-Δmuc. The overall higher binding force for the mutant virus translates into a smaller k_off_ (reproduced with permission from [98]). (C) Single particle tracking (SPT) with total internal reflection fluorescence (TIRF) microscopy is used to determine the diffusion coefficient of individual GAG-binding viruses. (i) The distribution of the diffusion coefficient is broad, spanning over several orders of magnitudes. (ii) Studies of the diffusion behavior (anomalous diffusion) of HSV-1 on different types of GAGs reveal that the diffusion coefficient depends on the type of GAG it is bound to (sHA, sulfated hyaluronic acid; CS, chondroitin sulfate; HS, heparan sulfate) (reproduced with permission from [100]). (D) Probing HSV-1 binding using native supported lipid bilayers (nSLBs). (i) To produce native supported lipid bilayers (nSLB), the cell is mechanically lysed and native membrane vesicles are isolated. These are then converted into hybrid vesicles via a sonication procedure in the presence of liposomes. The hybrid vesicles spontaneously form a nSLB on glass. The nSLBs were then used to probe the influence of HS on HSV-1 binding. Removal of HS through heparinase III treatment leads to (ii) a reduction in association rate constant (k_on_) and (iii) an increase in the dissociation rate constant (k_off_). Blue, untreated; red, heparinase treated; black, negative control. Reproduced with permission from [106, 101]

In an attempt to elucidate the mechanisms regulating virus-GAG interactions at the cell surface, minimal models of the cellular glycocalyx have been implemented. These typically consist of isolated GAGs, immobilized on a sensor surface in a biomimetic fashion [97–102]. For example, the immobilization of GAG chains in an end-on fashion via their reducing ends (Fig. 5A) mimics the presentation of the carbohydrates on the core proteins of proteoglycans. The advantage of such a bioanalytical platform is that it can be used to systematically investigate how the physicochemical properties of the GAG adlayers, including GAG type, chain length, degree of sulfation, and chain density, influence the characteristics of the interactions of viruses in the glycocalyx. Such a platform has been extensively implemented, for example, in the context of studying, on a molecular level, how attachment, detachment, and diffusion of HSVs are regulated at the cell surface [97, 98, 100, 103]. Specifically, the modulatory roles of the viral mucin-like region, a cluster of *O*-linked glycans, found on viral glycoprotein gC of HSV-1, and of the cellular GAGs have been explored. In a reductionist approach, the binding activity of isolated gC proteins to sensor-immobilized CS chains was investigated using a SPR-based binding assay [97]. A comparison of the binding behavior of wild-type gC-1 (gC-WT) and mucin-deleted gC-1 (gC-Δmuc) revealed that the apparent affinity of the mutant was significantly lower than that of the wild type, in spite of the fact that its dissociation was drastically slower. This indicated that gC-Δmuc attached less efficiently to the GAGs as compared to the wild-type gC protein, but that once formed, the gC-Δmuc-CS complex was more stable. Importantly, this result is in line with observations on the cellular level, which revealed that the mucin-deleted mutant virus is less sensitive to inhibition through GAG mimetics [97, 104], while having a reduced ability to detach from the surface of infected cells [97]. In addition to this, gC-WT was found to bind with similar affinity to CS and to an artificially sulfated GAG, sulfated hyaluronic acid (sHA), in spite of the lower sulfation density of the former. This suggests that binding is not merely mediated by electrostatic interaction with the negatively charged sulfates but that a certain degree of specificity is provided by the enzymatically sulfated native GAG, as compared to the artificial one, which is sulfated chemically in a random fashion [97]. These observations on the gC protein level were further corroborated by studying the binding behavior of individual virus particles in the GAG mimetic platform using TIRF microscopy. These studies further confirmed that viral particles with gC mutants, lacking the mucin-like region (HSV-1-gC-Δmuc), associate less efficiently to the carbohydrates than the HSV-1-WT [97]. Further molecular details of the detachment behavior of individual GAG-bound virus particles were obtained using AFM-based DFS (Fig. 5B). Such experiments performed with HSV-1-WT and HSV-1-gC-Δmuc revealed that deletion of the mucin-like region correlated with a ~ 6-fold decrease in *k*_off_ [98], once again in line with observations on the protein level [97]. Further data analysis also suggested that the mucin-like region modulates the interaction by regulating the affinity, type, and number of glycoproteins involved in virus-GAG interactions [98], most likely by initially shielding other viral glycoproteins, preventing them from engaging prematurely in an interaction with cell-surface receptors. Such a shielding effect has also been reported for other herpesviruses, such as the murine herpesvirus (MHV-4), where the heavily *O*-glycosylated glycoprotein gp150 was found to negatively regulate virus attachment to GAGs by reducing the number of contacts initially formed with GAGs, as revealed by AFM-based DFS performed with GAG-presenting surfaces. This observation was further confirmed by similar experiments on live cells, where the gp150-negative virus mutant was found to adhere with increased frequency to GAG-positive cells but not to GAG-negative ones [99]. In a different study, the diffusive behavior of GAG-bound viruses was investigated. Single particle tracking of GAG-bound HSV-1 particles using TIRF microscopy revealed that GAG-bound viruses have the capability of diffusing laterally on predominantly immobile GAG chains (Fig. 5C), presumably through a hopping or rolling behavior resulting from a molecular exchange mechanism where the formation and dissolution of individual bonds between viral glycoproteins and neighboring cellular GAGs allow for a lateral motion [100]. These results suggest that GAG-binding viruses such as HSV may take advantage of relatively weak interactions with GAGs to diffuse through the glycocalyx and along the cell surface, to reach the appropriate entry receptor. This hypothesis was recently further supported by observations on the cellular level (Bally et al. unpublished).

So far, we have illustrated how minimal cell surface mimics can be used to study virus-GAG interactions, with the example of HSV-1. In such a reductionist approach, only the molecules of interest (GAGs) are taken into consideration, allowing for systematic investigations of how selected components contribute to the overall virus membrane interactions. However, in some cases, a bioanalytical platform presenting the complete repertoire of the plasma membrane components, including lipids, carbohydrates, and proteins, in a native-like environment may be desirable. The use of such a platform may be beneficial for applications requiring a better understanding of the way the virus interacts with the membrane as a whole, i.e., with all receptors, co-receptors, and adhesion factors, both known and unknown. This may be useful for example in the context of efficiently screening for virus binding inhibitors. Additionally, a platform generated from cellular membrane extracts facilitates the study of membrane components difficult to isolate and purify. Along these lines, a number of research groups have proposed approaches to deposit native-membrane material purified from cells at a sensor surface, in the form of supported lipid bilayers of complex composition, i.e., native supported lipid bilayers (nSLBs). The advantage of such nSLB platforms is that they display the full compositional complexity of the cell surface, at a given time, but are decoupled from the physiological processes present in live cells. Accordingly, nSLBs have been implemented to virus research [58, 59, 101, 105], illustrating how they can be used to decouple the contribution of individual cell membrane components from the ones of other cellular factors, when studying virus-membrane interactions. The approach, originally presented by Pace at al. [106, 107] to produce compositionally complex nSLBs (Fig. 5D(i)), has recently been implemented to herpesvirus research [101]. Here, nSLBs were produced by lysing a cell of interest and harvesting plasma membrane material in the form of native membrane vesicles. These native membrane vesicles were then combined with synthetic lipids through sonication, yielding hybrid vesicles containing the original components of the cell but also increased amounts of phosphatidylcholine and pegylated lipids. These hybrid vesicles readily fuse into a supported lipid bilayer with preserved transmembrane protein fluidity [106] (Fig. 5D(i)). Using this nSLB platform and TIRF microscopy to visualize individual HSV-1 virus binding, it was reported that heparinase treatment leads to a decrease in the virus particle association rate and an increase in the dissociation rate by a factor of 1.6 and 2.5, respectively (Fig. 5D(ii and iii)), making this interaction more mobile. This analytical approach therefore provides quantitative insights into how heparan sulfate influences virus binding, release, and diffusion at the cell surface.

Taken together, this section illustrates the potential of working with bioanalytical platforms relying on cell surface mimics in constant feedback with in vitro cell-based assays to gain a mechanistic understanding, on the molecular level, of how cellular processes are regulated in live cells. It demonstrates how an experimental approach relying on combining various levels of molecular complexity can lead to drastically new insights into cell biology and virology.

### HIV as a model system to study protein receptor binding, fusion, and cellular entry

Currently, there is still an open debate on the relative importance of HIV-1 entry via fusion at the plasma membrane or fusion in the endosomes, and more importantly, about which pathways that lead to productive viral replication in the target cells. This controversy brings up fundamental questions about the individual and/or synergistic roles of the three major factors likely to influence HIV-1 fusion, i.e., interactions with receptors/co-receptors, lipid membrane factors (composition, domains, curvature, diffusion, etc.), and pH conditions within the endosomes.

Cell membrane mimics of increasing complexity have been used by the Tamm group to elucidate the mechanisms by which nanometer-sized domains rich in cholesterol and sphingomyelin facilitate fusion of HIV-1 with cellular membranes. Such investigations were motivated by reports indicating that the receptor and co-receptor for HIV-1 infection are preferentially enriched in lipid domains [108], implying that those domains are the sites of membrane fusion. This may however appear energetically unfavorable at first glance, since the rigidity and tighter lipid packing of cholesterol-rich domains may hinder membrane rupturing or fusion. To assess this issue and quantify energy barriers, studies were carried out on compositionally simple cell membrane mimics [63, 65]. These consisted of lipid-only membranes containing liquid-order(Lo) domains, mimicking the lipid composition of the plasma membrane of the target cells. This reductionist approach revealed that both the fusion peptide on HIV-1 gp41 and pseudotype HIV-1 particles interact predominantly at Lo/Ld(liquid-disordered) phase boundaries. Membrane fusion was also found to occur preferentially (~ 70%) in those areas, suggesting that the edges of lipid domains are the preferred sites of fusion and may thus play a key role as a site of HIV-1 entry [63]. Further investigations using various lipid compositions, as well as compounds reducing the line tension at Lo/Ld boundaries (linactants), reveal that reduction of line tension at the domain boundaries is a major energetic driver for HIV-1 fusion, and possibly entry [65].

**Fig. 6:**
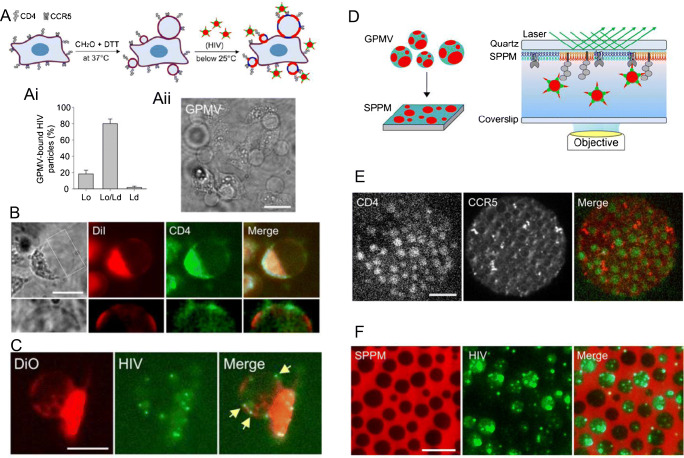
HIV-1 binding and fusion events observed using confocal fluorescence and TIRF microscopy. (A) Scheme showing cell membrane derived giant plasma membrane vesicle (GPMV) formation with lipid ordered (L_o_=red) and disordered (L_d_=blue) phases and HIV-1 binding preferentially to the L_o_/L_d_ boundaries. (Ai) Number of HIV-1 particles bound to L_o_, L_d_, and L_o_/L_d_. Corresponding representative confocal images are shown and described in panels below. (Aii) The bright-field image shows GPMVs formed and attached to cells. (B) Confocal micrographs of GPMVs depict how, at higher resolution of selected rectangle, CD4 (immunolabeled) distributes into L_o_ phase, separated from Dil staining (lipid anchoring dye, known to distribute into the L_d_ phase). (C) Fluorescent micrographs showing the distribution of HIV-1 (labeled green with mKO-Gag) at the single particle level binding to the L_o_ phase and possibly to L_o_/L_d_ boundaries but less to the L_d_ phase (labeled with DiO). (D) Scheme of formation of a supported planar plasma membrane (SPPM) and its application for dynamic imaging of HIV-1 binding and fusion using TIRF microscopy. (E) TIRF micrographs showing how differently CD4 distributes into the L_o_ phase while CCR5 tends to distribute at L_o_/L_d_ boundaries and in L_d_ phase. (F) TIRF images illustrating how (mKO-Gag) labeled HIV-1 particles bind to the SPPM (L_d_ phases labeled with Dil). It appears that the virus tends to bind in the L_o_ phase (green spots) and fuses at the L_o_/L_d_ boundaries (diffused green signal). Time-lapse TIRF images were further analyzed to determine the percentage of HIV-1 binding, hemifusion, and fusion events in L_o_, L_d_, and L_o/d_ boundaries of the SPPM. Reproduced with permission from [59]

Mechanistic studies of binding and fusion of HIV using reconstituted in vitro systems are complicated by the fact that the process requires two essential cellular transmembrane proteins: the receptor CD4 and a co-receptor, either CCR5 or CXCR4. To include receptors and co-receptors in their bioanalytical assays, the Tamm group used giant unilamellar vesicles and planar supported membranes derived directly from the plasma membranes of cells expressing CD4 and CCR5 [58, 59]. Giant plasma membrane vesicles (GPMVs) were obtained by gently treating the cells with formaldehyde and dithiothreitol, leading to the formation of membrane blebs (Fig. 6A). These blebs were converted into supported planar plasma membranes (SPPM) by fusing the GPMVs onto polymer supported lipid monolayers at an elevated temperature (>37°C). This resulted in a planar membrane geometry which facilitated the recording of single fusion events over large surface areas by TIRF microscopy (Fig. 6C). It also allowed for classification of the fusion events into hemifusion or complete fusion. Importantly, upon cooling to room temperature, these structures were found to exhibit large scale (μm size) phase separation, facilitating the study of processes occurring in lipid domains and at their boundaries. Immunofluorescence staining of receptor and co-receptor confirmed that CD4 co-localizes within the Lo phase, while the co-receptor CCR5 was distributed at the Lo/Ld phase boundaries. Moreover, in agreement with observations on the lipid-only systems, receptor-bound HIV-1 pseudotypes preferentially localized at the Lo/Ld boundary. Fusion also occurred at such boundaries. Furthermore, the presence of these boundaries was a prerequisite for successful fusion, as verified by disrupting the Lo domains by cyclodextrin treatment to remove cholesterol or using lysolipids to dissolve the microdomains [59]. Besides providing quantitative information on the fusion behavior of HIV pseudotypes on CD4^+^/CCR5^+^ membranes [59], such SPPMs were used to gain detailed mechanistic insights into the antiviral mode of action of the host restriction factors Serincs and their influence on the different steps of HIV-1 fusion [58].

While the bioanalytical platforms presented above allowed the examination of virus-membrane interactions at the single virus level and contributed to reveal the molecular mechanism of HIV fusion, they can neither be applied to evaluate the statistical distribution of different fusion or entry pathways nor to comment on whether they lead to cell infection. This can only be achieved by imaging infected cells and by visualizing the binding and entry of individual virus particles in a time-dependent manner and with a high spatial resolution. In the last two decades, a number of research groups have established single virus imaging conditions to study early and late stages of virus replication [4, 109–113]. Fluorescence imaging of HIV-1 attachment and fusion on a single particle level was extensively carried out recently by the Melikyan group [45, 111, 113] who established multi-color fluorescence imaging of single viruses to distinguish between plasma membrane fusion and endosomal fusion, and to evaluate the statistical distribution of the different fusion pathways. Moreover, they connected these fusion events to successful viral cell infection. Miyuachi K. et al. [45, 61] revealed three populations of HIV viruses. The first type of virus particles is relatively static at the plasma membrane and experiences early hemifusion but no release of the viral content. The other two types of virus particles are endocytosed and become mobile with or without directional transport towards the nucleus. Endosomal fusion was correlated with successful infection, using virus pseudotypes carrying a reporter gene. Furthermore, Miyuachi K. et al. [61] and Sood C. et al. [60] used a pH-sensitive tag at the virus surface to confirm that HIV-1 particles only release the viral content after entering acidic endosomes, and not at the plasma membrane. A similar imaging approach with quantitative evaluation showed that fusion of retrovirus homologues to HIV-1 also occurs in early or late endosomes [62]. The viral capsid, either intact or partially intact, was slowly released into the cytosol but only after the endosomal fusion, as confirmed upon imaging of dual-color labeled HIV-1 particles [60, 114]. In conclusion, the work by the Melikyan group illustrates that such co-localization imaging in a time-dependent manner is a reliable approach to analyze the statistical distribution of membrane fusion events and to follow the time evolution of the pre- to post-fusion process leading to release and replication of the viral genome.

## Conclusions and future perspective

Using several examples of well-characterized enveloped and non-enveloped viruses, this review highlights the nature of the information obtainable through implementation of emerging bioanalytical tools to study virus-membrane interactions. It illustrates the potential of such approaches in the context of studying viral binding kinetics, diffusivity, and fusion at the cell membrane, while emphasizing the importance of multivalency in virus binding and uptake.

The appearance of SARS-CoV-2, and the current global pandemic, has reminded us all of the importance of developing new vaccination platforms against viral diseases together with powerful antiviral drugs. In this context, it is our hope to have demonstrated that, beyond providing insights into the molecular mechanisms underpinning viral infection, emerging bioanalytical techniques also represent a solid foundation to the development of antivirals interfering with complex cellular entry pathways. This is exemplified in this review by the capacity to pinpoint how the neuraminidase inhibitor zanamivir affects IAV attachment, as well as the possibility to quantify the extent to which host molecules must be blocked before release of attached virus particles is initiated.

A variety of antiviral strategies targeting different aspects of interactions between viruses and cell membranes have already reached clinical trial or even been approved [114–116]. Examples include the HIV drugs maraviroc and fostemsavir which target a cellular co-receptor and a viral adhesion protein, respectively [114–116]; docosanol which has been suggested to act on HSV binding [114]; and the influenza drug DAS181 which prevents virus attachment through its sialidase activity [117]. Additionally, monoclonal antibodies, such as the ones against SARS-CoV-2, which have recently gained emergency authorization in several countries [118], are also a promising class of binding inhibitors, likely to see their implementation drastically increase in the coming years [114]. The techniques described in this review have the potential to provide further mechanistic insights into the mode of action of such antivirals. Thereby, they will likely contribute both to refining existing antiviral strategies and to proposing radically new approaches for targeted treatments.

The current bottleneck to the widespread implementation of the emerging bioanalytical methods described here is that they require advanced instrumentation which is not yet suitable for high-throughput screening. However, optical microscopy in particular is at present witnessing tremendous developmental gains making it very likely that many approaches discussed in this review will soon become broadly available even in the biosafety level 3 and 4 laboratories required to work with highly pathogenic viruses.

## References

[1] Marsh M, Helenius A (2006). Virus entry: open sesame. Cell..

[2] Mercer J, Schelhaas M, Helenius A (2010). Virus entry by endocytosis. Annu Rev Biochem.

[3] Helenius A (2018). Virus entry: looking back and moving forward. J Mol Biol.

[4] Chernomordik LV, Kozlov MM (2005). Membrane hemifusion: crossing a chasm in two leaps. Cell..

[5] Tang T, Bidon M, Jaimes JA, Whittaker GR, Daniel S. Coronavirus membrane fusion mechanism offers as a potential target for antiviral development. Antivir Res. 2020;104792. 10.1016/j.antiviral.2020.104792 PMC7194977 32272173

[6] White JM, Delos SE, Brecher M, Schornberg K (2008). Structures and mechanisms of viral membrane fusion proteins: multiple variations on a common theme. Crit Rev Biochem Mol Biol.

[7] Mammen M, Choi SK, Whitesides GM (1998). Polyvalent interactions in biological systems: implications for design and use of multivalent ligands and inhibitors. Angew Chem Int Ed Eng.

[8] Olofsson S, Bergstrom T (2005). Glycoconjugate glycans as viral receptors. Ann Med.

[9] Thompson AJ, de Vries RP, Paulson JC (2019). Virus recognition of glycan receptors. Curr Opin Virol.

[10] Stroh LJ, Stehle T (2014). Glycan engagement by viruses: receptor switches and specificity. Annu Rev Virol.

[11] Kamhi E, Joo EJ, Dordick JS, Linhardt RJ (2013). Glycosaminoglycans in infectious disease. Biol Rev Camb Philos Soc.

[12] Martinez-Veracoechea FJ, Frenkel D (2011). Designing super selectivity in multivalent nano-particle binding. Proc Natl Acad Sci U S A.

[13] Fasting C, Schalley CA, Weber M, Seitz O, Hecht S, Koksch B, Dernedde J, Graf C, Knapp EW, Haag R (2012). Multivalency as a chemical organization and action principle. Angew Chem Int Ed Eng.

[14] Ewers H, Romer W, Smith AE, Bacia K, Dmitrieff S, Chai W, Mancini R, Kartenbeck J, Chambon V, Berland L, Oppenheim A, Schwarzmann G, Feizi T, Schwille P, Sens P, Helenius A, Johannes L (2010). GM1 structure determines SV40-induced membrane invagination and infection. Nat Cell Biol.

[15] Neu U, Woellner K, Gauglitz G, Stehle T (2008). Structural basis of GM1 ganglioside recognition by simian virus 40. Proc Natl Acad Sci U S A.

[16] Tsai B, Gilbert JM, Stehle T, Lencer W, Benjamin TL, Rapoport TA (2003). Gangliosides are receptors for murine polyoma virus and SV40. EMBO J.

[17] Campanero-Rhodes MA, Smith A, Chai W, Sonnino S, Mauri L, Childs RA, Zhang Y, Ewers H, Helenius A, Imberty A, Feizi T (2007). N-glycolyl GM1 ganglioside as a receptor for simian virus 40. J Virol.

[18] Varki A (2009). Multiple changes in sialic acid biology during human evolution. Glycoconj J.

[19] Poulin DL, DeCaprio JA (2006). Is there a role for SV40 in human cancer?. J Clin Oncol.

[20] Banyai K, Estes MK, Martella V, Parashar UD (2018). Viral gastroenteritis. Lancet..

[21] Prasad BV, Hardy ME, Dokland T, Bella J, Rossmann MG, Estes MK (1999). X-ray crystallographic structure of the Norwalk virus capsid. Science..

[22] Rydell GE, Kindberg E, Larson G, Svensson L (2011). Susceptibility to winter vomiting disease: a sweet matter. Rev Med Virol.

[23] Rimkute I, Thorsteinsson K, Henricsson M, Tenge VR, Yu X, Lin SC, Haga K, Atmar RL, Lycke N, Nilsson J, Estes MK, Bally M, Larson G (2020). Histo-blood group antigens of glycosphingolipids predict susceptibility of human intestinal enteroids to norovirus infection. J Biol Chem.

[24] Tamura M, Natori K, Kobayashi M, Miyamura T, Takeda N (2004). Genogroup II noroviruses efficiently bind to heparan sulfate proteoglycan associated with the cellular membrane. J Virol.

[25] Rydell GE, Nilsson J, Rodriguez-Diaz J, Ruvoen-Clouet N, Svensson L, Le Pendu J, Larson G (2009). Human noroviruses recognize sialyl Lewis x neoglycoprotein. Glycobiology..

[26] Han L, Tan M, Xia M, Kitova EN, Jiang X, Klassen JS (2014). Gangliosides are ligands for human noroviruses. J Am Chem Soc.

[27] Bally M, Rydell GE, Zahn R, Nasir W, Eggeling C, Breimer ME, Svensson L, Hook F, Larson G (2012). Norovirus GII.4 virus-like particles recognize galactosylceramides in domains of planar supported lipid bilayers. Angew Chem Int Ed Eng.

[28] de Graaf M, Fouchier RA (2014). Role of receptor binding specificity in influenza A virus transmission and pathogenesis. EMBO J.

[29] Byrd-Leotis L, Cummings RD, Steinhauer DA. The interplay between the host receptor and influenza virus hemagglutinin and neuraminidase. Int J Mol Sci. 2017;18(7). 10.3390/ijms18071541. 10.3390/ijms18071541 PMC5536029 28714909

[30] Wagner R, Matrosovich M, Klenk HD (2002). Functional balance between haemagglutinin and neuraminidase in influenza virus infections. Rev Med Virol.

[31] Thompson AJ, Paulson JC. Adaptation of influenza viruses to human airway receptors. J Biol Chem. 2020. 10.1074/jbc.REV120.013309. 10.1074/jbc.REV120.013309 PMC7948470 33144323

[32] Byrd-Leotis L, Jia N, Dutta S, Trost JF, Gao C, Cummings SF, Braulke T, Muller-Loennies S, Heimburg-Molinaro J, Steinhauer DA, Cummings RD (2019). Influenza binds phosphorylated glycans from human lung. Sci Adv.

[33] Fatahzadeh M, Schwartz RA (2007). Human herpes simplex virus infections: epidemiology, pathogenesis, symptomatology, diagnosis, and management. J Am Acad Dermatol.

[34] Grunewald K, Desai P, Winkler DC, Heymann JB, Belnap DM, Baumeister W, Steven AC (2003). Three-dimensional structure of herpes simplex virus from cryo-electron tomography. Science..

[35] Hilterbrand AT, Heldwein EE (2019). Go go gadget glycoprotein!: HSV-1 draws on its sizeable glycoprotein tool kit to customize its diverse entry routes. PLoS Pathog.

[36] Llorente Garcia I, Marsh M (1862). A biophysical perspective on receptor-mediated virus entry with a focus on HIV. Biochim Biophys Acta Biomembr.

[37] Connell BJ, Lortat-Jacob H (2013). Human immunodeficiency virus and heparan sulfate: from attachment to entry inhibition. Front Immunol.

[38] Carneiro FA, Bianconi ML, Weissmuller G, Stauffer F, Da Poian AT (2002). Membrane recognition by vesicular stomatitis virus involves enthalpy-driven protein-lipid interactions. J Virol.

[39] Schon A, Madani N, Klein JC, Hubicki A, Ng D, Yang X, Smith AB, Sodroski J, Freire E (2006). Thermodynamics of binding of a low-molecular-weight CD4 mimetic to HIV-1 gp120. Biochemistry..

[40] Sauter NK, Bednarski MD, Wurzburg BA, Hanson JE, Whitesides GM, Skehel JJ, Wiley DC (1989). Hemagglutinins from two influenza virus variants bind to sialic acid derivatives with millimolar dissociation constants: a 500-MHz proton nuclear magnetic resonance study. Biochemistry..

[41] Mallagaray A, Creutznacher R, Dulfer J, Mayer PHO, Grimm LL, Orduna JM, Trabjerg E, Stehle T, Rand KD, Blaum BS, Uetrecht C, Peters T (2019). A post-translational modification of human norovirus capsid protein attenuates glycan binding. Nat Commun.

[42] Szklarczyk OM, Gonzalez-Segredo N, Kukura P, Oppenheim A, Choquet D, Sandoghdar V, Helenius A, Sbalzarini IF, Ewers H (2013). Receptor concentration and diffusivity control multivalent binding of Sv40 to membrane bilayers. PLoS Comput Biol.

[43] Shi J, Yang T, Kataoka S, Zhang Y, Diaz AJ, Cremer PS (2007). GM1 clustering inhibits cholera toxin binding in supported phospholipid membranes. J Am Chem Soc.

[44] Liese S, Netz RR (2018). Quantitative prediction of multivalent ligand-receptor binding affinities for influenza, cholera, and anthrax inhibition. ACS Nano.

[45] Miyauchi K, Kim Y, Latinovic O, Morozov V, Melikyan GB (2009). HIV enters cells via endocytosis and dynamin-dependent fusion with endosomes. Cell..

[46] Janshoff A, Galla HJ, Steinem C (2000). Piezoelectric mass-sensing devices as biosensors-an alternative to optical biosensors?. Angew Chem Int Ed Eng.

[47] Hook F, Kasemo B, Nylander T, Fant C, Sott K, Elwing H (2001). Variations in coupled water, viscoelastic properties, and film thickness of a Mefp-1 protein film during adsorption and cross-linking: a quartz crystal microbalance with dissipation monitoring, ellipsometry, and surface plasmon resonance study. Anal Chem.

[48] Reviakine I, Johannsmann D, Richter RP (2011). Hearing what you cannot see and visualizing what you hear: interpreting quartz crystal microbalance data from solvated interfaces. Anal Chem.

[49] Jung LS, Campbell CT, Chinowsky TM, Mar MN, Yee SS (1998). Quantitative interpretation of the response of surface plasmon resonance sensors to adsorbed films. Langmuir..

[50] Rupert DLM, Shelke GV, Emilsson G, Claudio V, Block S, Lasser C, Dahlin A, Lotvall JO, Bally M, Zhdanov VP, Hook F (2016). Dual-wavelength surface plasmon resonance for determining the size and concentration of sub-populations of extracellular vesicles. Anal Chem.

[51] Rydell GE, Dahlin AB, Hook F, Larson G (2009). QCM-D studies of human norovirus VLPs binding to glycosphingolipids in supported lipid bilayers reveal strain-specific characteristics. Glycobiology..

[52] Nasir W, Nilsson J, Olofsson S, Bally M, Rydell GE (2014). Parvovirus B19 VLP recognizes globoside in supported lipid bilayers. Virology..

[53] Axelrod D (1989). Total internal reflection fluorescence microscopy. Methods Cell Biol.

[54] Gunnarsson A, Dexlin L, Wallin P, Svedhem S, Jonsson P, Wingren C, Hook F (2011). Kinetics of ligand binding to membrane receptors from equilibrium fluctuation analysis of single binding events. J Am Chem Soc.

[55] Bally M, Gunnarsson A, Svensson L, Larson G, Zhdanov VP, Hook F. Interaction of single viruslike particles with vesicles containing glycosphingolipids. Phys Rev Lett. 2011;107(18) doi:ARTN 18810310.1103/PhysRevLett.107.188103. 10.1103/PhysRevLett.107.188103 22107678

[56] Merkel R, Nassoy P, Leung A, Ritchie K, Evans E (1999). Energy landscapes of receptor-ligand bonds explored with dynamic force spectroscopy. Nature..

[57] Evans E (2001). Probing the relation between force--lifetime--and chemistry in single molecular bonds. Annu Rev Biophys Biomol Struct.

[58] Ward AE, Kiessling V, Pornillos O, White JM, Ganser-Pornillos BK, Tamm LK (2020). HIV-cell membrane fusion intermediates are restricted by Serincs as revealed by cryo-electron and TIRF microscopy. J Biol Chem.

[59] Yang ST, Kreutzberger AJB, Kiessling V, Ganser-Pornillos BK, White JM, Tamm LK. HIV virions sense plasma membrane heterogeneity for cell entry. Sci Adv. 2017;3(6) doi:ARTN e170033810.1126/sciadv.1700338. 10.1126/sciadv.1700338 PMC5489272 28782011

[60] Sood C, Marin M, Mason CS, Melikyan GB (2016). Visualization of content release from cell surface-attached single HIV-1 particles carrying an extra-viral fluorescent pH-sensor. PLoS One.

[61] Miyauchi K, Marin M, Melikyan GB (2011). Visualization of retrovirus uptake and delivery into acidic endosomes. Biochem J.

[62] Padilla-Parra S, Matos PM, Kondo N, Marin M, Santos NC, Melikyan GB (2012). Quantitative imaging of endosome acidification and single retrovirus fusion with distinct pools of early endosomes. Proc Natl Acad Sci U S A.

[63] Yang ST, Kiessling V, Simmons JA, White JM, Tamm LK (2015). HIV gp41-mediated membrane fusion occurs at edges of cholesterol-rich lipid domains. Nat Chem Biol.

[64] Rawle RJ, Boxer SG, Kasson PM (2016). Disentangling viral membrane fusion from receptor binding using synthetic DNA-lipid conjugates. Biophys J.

[65] Yang ST, Kiessling V, Tamm LK. Line tension at lipid phase boundaries as driving force for HIV fusion peptide-mediated fusion. Nat Commun. 2016;7 doi:ARTN 1140110.1038/ncomms11401. 10.1038/ncomms11401 PMC4853434 27113279

[66] Floyd DL, Ragains JR, Skehel JJ, Harrison SC, van Oijen AM (2008). Single-particle kinetics of influenza virus membrane fusion. Proc Natl Acad Sci U S A.

[67] Costello DA, Lee DW, Drewes J, Vasquez KA, Kisler K, Wiesner U, Pollack L, Whittaker GR, Daniel S (2012). Influenza virus-membrane fusion triggered by proton uncaging for single particle studies of fusion kinetics. Anal Chem.

[68] Wessels L, Elting MW, Scimeca D, Weninger K (2007). Rapid membrane fusion of individual virus particles with supported lipid bilayers. Biophys J.

[69] Baumgärtel V, Ivanchenko S, Dupont A, Sergeev M, Wiseman PW, Kräusslich H-G, Bräuchle C, Müller B, Lamb DC (2011). Live-cell visualization of dynamics of HIV budding site interactions with an ESCRT component. Nat Cell Biol.

[70] Ewers H, Smith AE, Sbalzarini IF, Lilie H, Koumoutsakos P, Helenius A (2005). Single-particle tracking of murine polyoma virus-like particles on live cells and artificial membranes. Proc Natl Acad Sci U S A.

[71] Favard C, Chojnacki J, Merida P, Yandrapalli N, Mak J, Eggeling C, Muriaux D (2019). HIV-1 Gag specifically restricts PI(4,5)P2 and cholesterol mobility in living cells creating a nanodomain platform for virus assembly. Sci Adv.

[72] Henderson HI, Hope TJ (2006). The temperature arrested intermediate of virus-cell fusion is a functional step in HIV infection. Virol J.

[73] Sandalon Z, Oppenheim A (1997). Self-assembly and protein-protein interactions between the SV40 capsid proteins produced in insect cells. Virology.

[74] Jiang X, Wang M, Graham DY, Estes MK (1992). Expression, self-assembly, and antigenicity of the Norwalk virus capsid protein. J Virol.

[75] Parveen N, Block S, Zhdanov VP, Rydell GE, Hook F (2017). Detachment of membrane bound virions by competitive ligand binding induced receptor depletion. Langmuir..

[76] Janshoff A, Steinem C, Sieber M, el Baya A, Schmidt MA, Galla HJ (1997). Quartz crystal microbalance investigation of the interaction of bacterial toxins with ganglioside containing solid supported membranes. Eur Biophys J Biophys Lett.

[77] Margheri G, D’Agostino R, Trigari S, Sottini S, Del Rosso M (2014). The beta-subunit of cholera toxin has a high affinity for ganglioside GM1 embedded into solid supported lipid membranes with a lipid raft-like composition. Lipids..

[78] Parveen N, Rydell GE, Larson G, Hytonen VP, Zhdanov VP, Hook F, Block S (2019). Competition for membrane receptors: norovirus detachment via lectin attachment. J Am Chem Soc.

[79] Parveen N, Rimkute I, Block S, Rydell GE, Midtvedt D, Larson G, Hytonen VP, Zhdanov VP, Lundgren A, Hook F (2018). Membrane deformation induces clustering of norovirus bound to glycosphingolipids in a supported cell-membrane mimic. J Phys Chem Lett.

[80] Rydell GE, Svensson L, Larson G, Johannes L, Romer W (2013). Human GII.4 norovirus VLP induces membrane invaginations on giant unilamellar vesicles containing secretor gene dependent alpha1,2-fucosylated glycosphingolipids. Biochim Biophys Acta.

[81] Evans E, Sackmann E (1988). Translational and rotational drag coefficients for a disk moving in a liquid membrane-associated with a rigid substrate. J Fluid Mech.

[82] Block S, Zhdanov VP, Hook F (2016). Quantification of multivalent interactions by tracking single biological nanoparticle mobility on a lipid membrane. Nano Lett.

[83] Block S. Brownian motion at lipid membranes: a comparison of hydrodynamic models describing and experiments quantifying diffusion within lipid bilayers. Biomolecules. 2018;8(2) doi:ARTN 3010.3390/biom8020030. 10.3390/biom8020030 PMC6023006 29789471

[84] Sieben C, Kappel C, Zhu R, Wozniak A, Rankl C, Hinterdorfer P, Grubmuller H, Herrmann A (2012). Influenza virus binds its host cell using multiple dynamic interactions. Proc Natl Acad Sci U S A.

[85] Muller M, Lauster D, Wildenauer HHK, Herrmann A, Block S (2019). Mobility-based quantification of multivalent virus-receptor interactions: new insights into influenza A virus binding mode. Nano Lett.

[86] Hidari KI, Shimada S, Suzuki Y, Suzuki T (2007). Binding kinetics of influenza viruses to sialic acid-containing carbohydrates. Glycoconj J.

[87] Watowich SJ, Skehel JJ, Wiley DC (1994). Crystal structures of influenza virus hemagglutinin in complex with high-affinity receptor analogs. Structure..

[88] Guo H, Rabouw H, Slomp A, Dai M, van der Vegt F, van Lent JWM, McBride R, Paulson JC, de Groot RJ, van Kuppeveld FJM, de Vries E, de Haan CAM (2018). Kinetic analysis of the influenza A virus HA/NA balance reveals contribution of NA to virus-receptor binding and NA-dependent rolling on receptor-containing surfaces. PLoS Pathog.

[89] Childs RA, Palma AS, Wharton S, Matrosovich T, Liu Y, Chai W, Campanero-Rhodes MA, Zhang Y, Eickmann M, Kiso M, Hay A, Matrosovich M, Feizi T (2009). Receptor-binding specificity of pandemic influenza A (H1N1) 2009 virus determined by carbohydrate microarray. Nat Biotechnol.

[90] McBride R, Paulson JC, de Vries RP. A miniaturized glycan microarray assay for assessing avidity and specificity of influenza A virus hemagglutinins. J Vis Exp. 2016;111. 10.3791/53847. 10.3791/53847 PMC4927745 27284789

[91] Lee DW, Hsu HL, Bacon KB, Daniel S (2016). Image restoration and analysis of influenza virions binding to membrane receptors reveal adhesion-strengthening kinetics. PLoS One.

[92] Cuellar-Camacho JL, Bhatia S, Reiter-Scherer V, Lauster D, Liese S, Rabe JP, Herrmann A, Haag R (2020). Quantification of multivalent interactions between sialic acid and influenza A virus spike proteins by single-molecule force spectroscopy. J Am Chem Soc.

[93] Wallert M, Nie C, Anilkumar P, Abbina S, Bhatia S, Ludwig K, Kizhakkedathu JN, Haag R, Block S (2020). Mucin-inspired, high molecular weight virus binding inhibitors show biphasic binding behavior to influenza A viruses. Small..

[94] Huang ML, Cohen M, Fisher CJ, Schooley RT, Gagneux P, Godula K (2015). Determination of receptor specificities for whole influenza viruses using multivalent glycan arrays. Chem Commun (Camb).

[95] Shukla D, Spear PG (2001). Herpesviruses and heparan sulfate: an intimate relationship in aid of viral entry. J Clin Invest.

[96] Reitsma S, Slaaf DW, Vink H, van Zandvoort MA (2007). oude Egbrink MG. The endothelial glycocalyx: composition, functions, and visualization. Pflugers Arch.

[97] Altgarde N, Eriksson C, Peerboom N, Phan-Xuan T, Moeller S, Schnabelrauch M, Svedhem S, Trybala E, Bergstrom T, Bally M (2015). Mucin-like region of herpes simplex virus type 1 attachment protein glycoprotein C (gC) modulates the virus-glycosaminoglycan interaction. J Biol Chem.

[98] Delguste M, Peerboom N, Le Brun G, Trybala E, Olofsson S, Bergstrom T, Alsteens D, Bally M (2019). Regulatory mechanisms of the mucin-like region on herpes simplex virus during cellular attachment. ACS Chem Biol.

[99] Delguste M, Zeippen C, Machiels B, Mast J, Gillet L, Alsteens D. Multivalent binding of herpesvirus to living cells is tightly regulated during infection. Sci Adv. 2018;4(8) doi:ARTN eaat127310.1126/sciadv.aat1273. 10.1126/sciadv.aat1273 PMC6097811 30128355

[100] Peerboom N, Block S, Altgarde N, Wahlsten O, Moller S, Schnabelrauch M, Trybala E, Bergstrom T, Bally M (2017). Binding kinetics and lateral mobility of HSV-1 on end-grafted sulfated glycosaminoglycans. Biophys J.

[101] Peerboom N, Schmidt E, Trybala E, Block S, Bergstrom T, Pace HP, Bally M (2018). Cell membrane derived platform to study virus binding kinetics and diffusion with single particle sensitivity. ACS Infect Dis.

[102] Rux AH, Lou H, Lambris JD, Friedman HM, Eisenberg RJ, Cohen GH (2002). Kinetic analysis of glycoprotein C of herpes simplex virus types 1 and 2 binding to heparin, heparan sulfate, and complement component C3b. Virology..

[103] Trybala E, Peerboom N, Adamiak B, Krzyzowska M, Liljeqvist JA, Bally M, et al. Herpes simplex virus type 2 mucin-like glycoprotein mgG promotes virus release from the surface of infected cells. Viruses. 2021;13(5). 10.3390/v13050887. 10.3390/v13050887 PMC8150390 34065826

[104] Ekbad M, Adarmak B, Bergefall K, Nenonen H, Roth A, Bergstrom T, Ferro V, Trybala E (2007). Molecular basis for resistance of herpes simplex virus type 1 mutants to the sulfated oligosaccharide inhibitor PI-88. Virology..

[105] Costello DA, Millet JK, Hsia CY, Whittaker GR, Daniel S (2013). Single particle assay of coronavirus membrane fusion with proteinaceous receptor-embedded supported bilayers. Biomaterials..

[106] Pace H, Nystrom LS, Gunnarsson A, Eck E, Monson C, Geschwindner S, Snijder A, Hook F (2015). Preserved transmembrane protein mobility in polymer-supported lipid bilayers derived from cell membranes. Anal Chem.

[107] Pace HP, Hannestad JK, Armonious A, Adamo M, Agnarsson B, Gunnarsson A, Micciulla S, Sjovall P, Gerelli Y, Hook F (2018). Structure and composition of native membrane derived polymer-supported lipid bilayers. Anal Chem.

[108] Waheed AA, Freed EO (2009). Lipids and membrane microdomains in HIV-1 replication. Virus Res.

[109] Brandenburg B, Zhuang X (2007). Virus trafficking – learning from single-virus tracking. Nat Rev Microbiol.

[110] Daecke J, Fackler OT, Dittmar MT, Kräusslich H-G (2005). Involvement of clathrin-mediated endocytosis in human immunodeficiency virus type 1 entry. J Virol.

[111] Melikyan GB (2014). HIV entry: a game of hide-and-fuse?. Current opinion in virology.

[112] Parveen N, Borrenberghs D, Rocha S, Hendrix J (2018). Single viruses on the fluorescence microscope: imaging molecular mobility, interactions and structure sheds new light on viral replication. Viruses..

[113] Sood C, Francis AC, Desai TM, Melikyan GB (2017). An improved labeling strategy enables automated detection of single-virus fusion and assessment of HIV-1 protease activity in single virions. J Biol Chem.

[114] De Clercq E, Li G (2016). Approved antiviral drugs over the past 50 years. Clin Microbiol Rev.

[115] Dorr P, Westby M, Dobbs S, Griffin P, Irvine B, Macartney M, Mori J, Rickett G, Smith-Burchnell C, Napier C, Webster R, Armour D, Price D, Stammen B, Wood A, Perros M (2005). Maraviroc (UK-427,857), a potent, orally bioavailable, and selective small-molecule inhibitor of chemokine receptor CCR5 with broad-spectrum anti-human immunodeficiency virus type 1 activity. Antimicrob Agents Chemother.

[116] Li G, Jing X, Zhang P, de Clercq E. Antiviral classification. In: Bamford DH, Zuckerman M, editors. Encyclopedia of virology. 4th ed: Elsevier; 2021. p. 121–30.

[117] Mazzon M, Marsh M. Targeting viral entry as a strategy for broad-spectrum antivirals. F1000Res. 2019;8. 10.12688/f1000research.19694.1. 10.12688/f1000research.19694.1 PMC6743247 31559009

[118] The COVID-19 Treatment Guidelines Panel’s Statement on the Emergency Use Authorizations of Anti-SARS-CoV-2 Monoclonal Antibodies for the Treatment of COVID-19. 2021. https://www.covid19treatmentguidelines.nih.gov/therapies/statement-on-anti-sars-cov-2-monoclonal-antibodies-eua/. Accessed 17 June 2021

